# New local magnitude scales for Egypt

**DOI:** 10.1038/s41598-024-80995-x

**Published:** 2024-12-23

**Authors:** Sherif M. Elhady, Mohamed Ezzelarab, M. Sami Soliman, Hussein S. Abdullah, Iman F. Abu El Nader, Ashraf Adly, Tharwat H. Abd-Elhafeez

**Affiliations:** 1 Egyptian National Seismic Network (ENSN), Department of Seismology, National Research Institute of Astronomy and Geophysics, Cairo, Egypt; 2 Geology Department, Faculty of Science, Al-Azhar University, Cairo, Egypt

**Keywords:** Egypt, Local magnitude scale, Wood–Anderson, Trilinear regression, Geophysics, Seismology

## Abstract

Local magnitude (M_L_) scales have been developed for Egypt based on 14,453 normalized Wood–Anderson amplitudes from 1670 earthquakes. These events were recorded by at least four seismic stations, with hypocentral depths of less than 40 km and earthquake magnitudes ranging from 0.1 to 6.5 M_L_. The dataset was processed using recordings from the two horizontal components. Egypt was divided into four sub-tectonic regions: South Egypt, North Egypt, the Red Sea, and the Mediterranean Sea. The decay of amplitudes with distance was examined across the entire dataset, providing attenuation characteristics segmented into three sections, with transition distances at 90 km and 175 km. Within each sub-tectonic region, the coefficients of the distance correction term (− log A0) and the station correction term (S) were determined using a trilinear geometrical scattering model and singular value decomposition, respectively. The newly derived relationship for Egypt has resulted in unbiased ML magnitude estimates over a substantial distance range (10–1000 km), thus ensuring consistent magnitude estimates from the ENSN network.

**Supplementary Information:**

The online version contains supplementary material available at 10.1038/s41598-024-80995-x.

## Introduction

The earthquake magnitude is used as a proxy to characterize the associated amount of energy released from an earthquake and has a direct relationship to its severity level. It is one of the main parameters for describing an earthquake. In the field of the observational seismology, there are numerous types of magnitude scales. Some of them are developed based on the logarithm of the amplitude of a particular phase; (a) the body wave magnitude (*mb*) proposed by Gutenberg^1^ based on body waves with period range 0.5–12 s, (b) the surface wave magnitude (*Ms*) based on about 20 s surface waves^2^ and it used to measure the earthquake size for shallow and distant earthquakes. Furthermore, other magnitude scales are independent of amplitude, such as the coda duration magnitude (*Md*) and the moment magnitude (*Mw*) scale. *Md* is based mainly on the duration of the seismic signal^3^ and *Mw* is based on the log of the seismic moment of the earthquake that is directly related to earthquake source physics^4^. *Md* scale is used to estimate the magnitude of local earthquakes as for the data recorded by USGS in Central California microearthquake network. *Mw* scale has the advantage that it does not saturate for large magnitudes. It is commonly used in seismic hazard studies. The most common one is the local magnitude scale (M_L_). It is an essential factor in observational seismology due to the convenience of computation in real-time^5^.

The concept of earthquake magnitude M_L_ is first introduced by Richter^6^ based on the logarithm of maximum amplitude (in millimetres) measured on a horizontal-component ground motion of Wood-Anderson and the distance correction term ($\:-log\left(Ao\right)\:$presented as a tabulated values to compensate the attenuation and unify the same magnitude value for one earthquake at all distances. A while after that, Hutton and Boor^7^ develop the ML scale relationship for southern California to include station correction term and number of generic constants. The standard formula of Hutton and Boore’s is applied in many regions all over the world and it is noticed that these constants are controlled by the attenuation behavior of the seismic waves, which varies from one region to another based on the geology of the region.

Many studies have been carried out to develop the local magnitude scales for specific regions. For instance, Brazier et al.^8^ developed the ML Scale for Ethiopian Plateau based on the inversion of 3218 corrected maximum S-wave amplitudes. Bajc et al.^9^ used the iterative least-square method to determine the attenuation function and station-component corrections and present the local magnitude scale for Slovenia. Scordilis et al.^10^ used the records of 98 digital broad-band stations to calculate a refined geometrical spreading factor and an anelastic attenuation coefficient for estimating local magnitudes in Greece. Similarly, Di Bona^11^ analysed the seismic signals recorded by the dense broadband network in Italy and developed the M_L_ Scale for crustal earthquakes in Italy. The resent studies^12–14^ flow the regional attenuation of seismic waves and describe the distance correction term in form of trilinear function.

The current article aims to develop local magnitude scales for Egypt in form of trilinear function. The abundant instrumental earthquake records in Egypt in addition to the heterogeneous geological setting motivate the authors to present new local magnitude scales for Egypt.

**Fig. 1:**
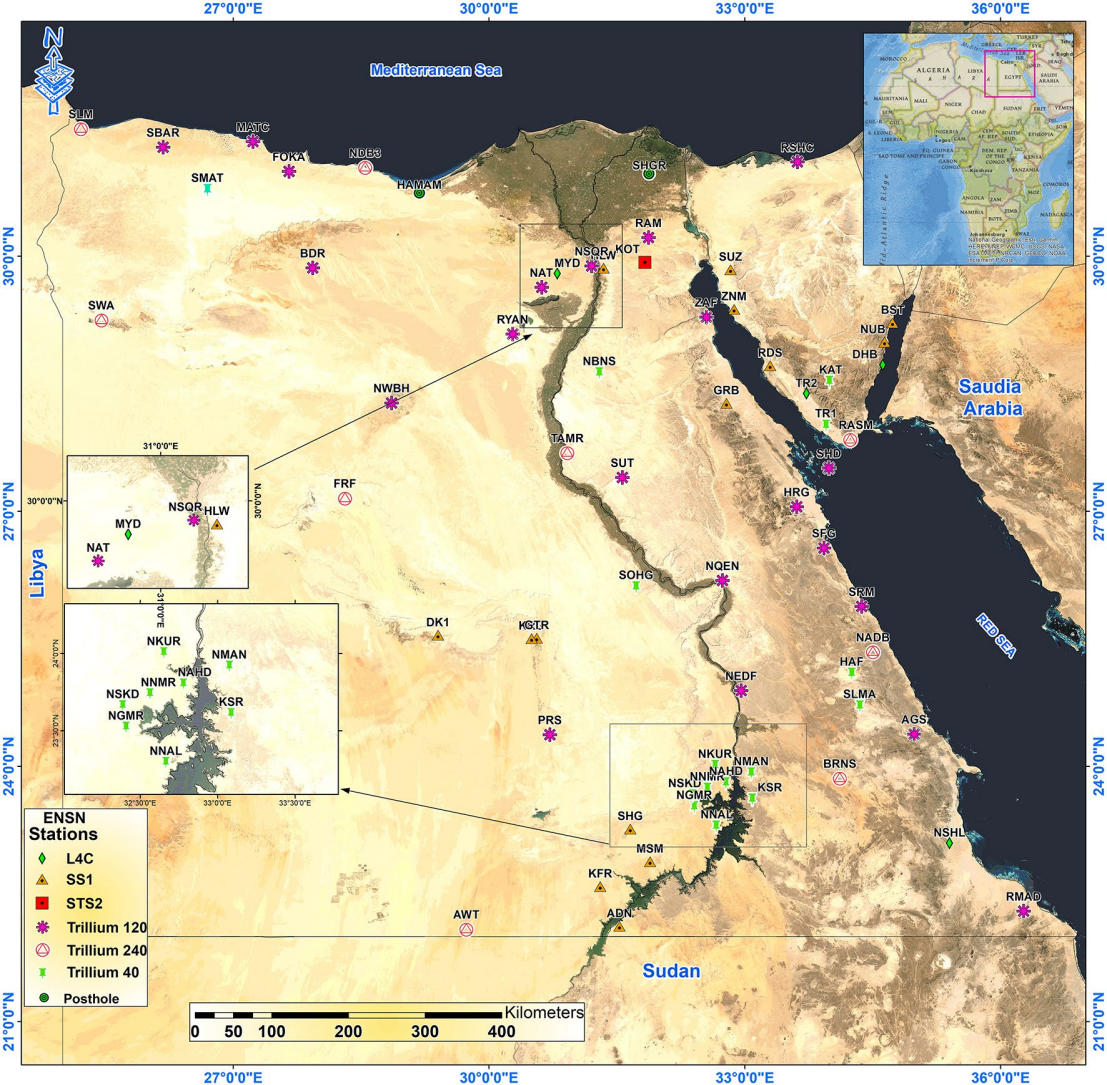
Distribution of weak motion stations, Egyptian National Seismological Network (ENSN) stations. This map was created by using ArcGIS 10.8.2 software and basemap produced by Ref.^16^.

It is worth noting that, the Egyptian National Seismic Network (ENSN), consisting of 63 seismic stations (Fig. 1), reported the local earthquake sizes from 1997 up to 2008 based on the relationship of the Richter^15^ and, for regional events, on Nuttli^12^. Nuttli introduced a magnitude scale (MN) designed explicitly for earthquakes in the eastern United States using 1-second Lg amplitudes recorded on short-period narrow-band vertical seismograms.

Since 2008, M_L_ magnitudes reported by the Egyptian National Seismic Network (ENSN) are based upon Alsaker et al.^17^ for a distance range of 10–200 km and up on Hutton and Boore^7^ for a distance range of 200–600 km.

Recently Abdullah et al.^18^ presented a M_L_ scale for the southern part of Egypt based on the records of earthquakes by the Egyptian National Seismic Network (ENSN) in the magnitude range 1.0–4.5.

## Seismotectonic framework

**Fig. 2:**
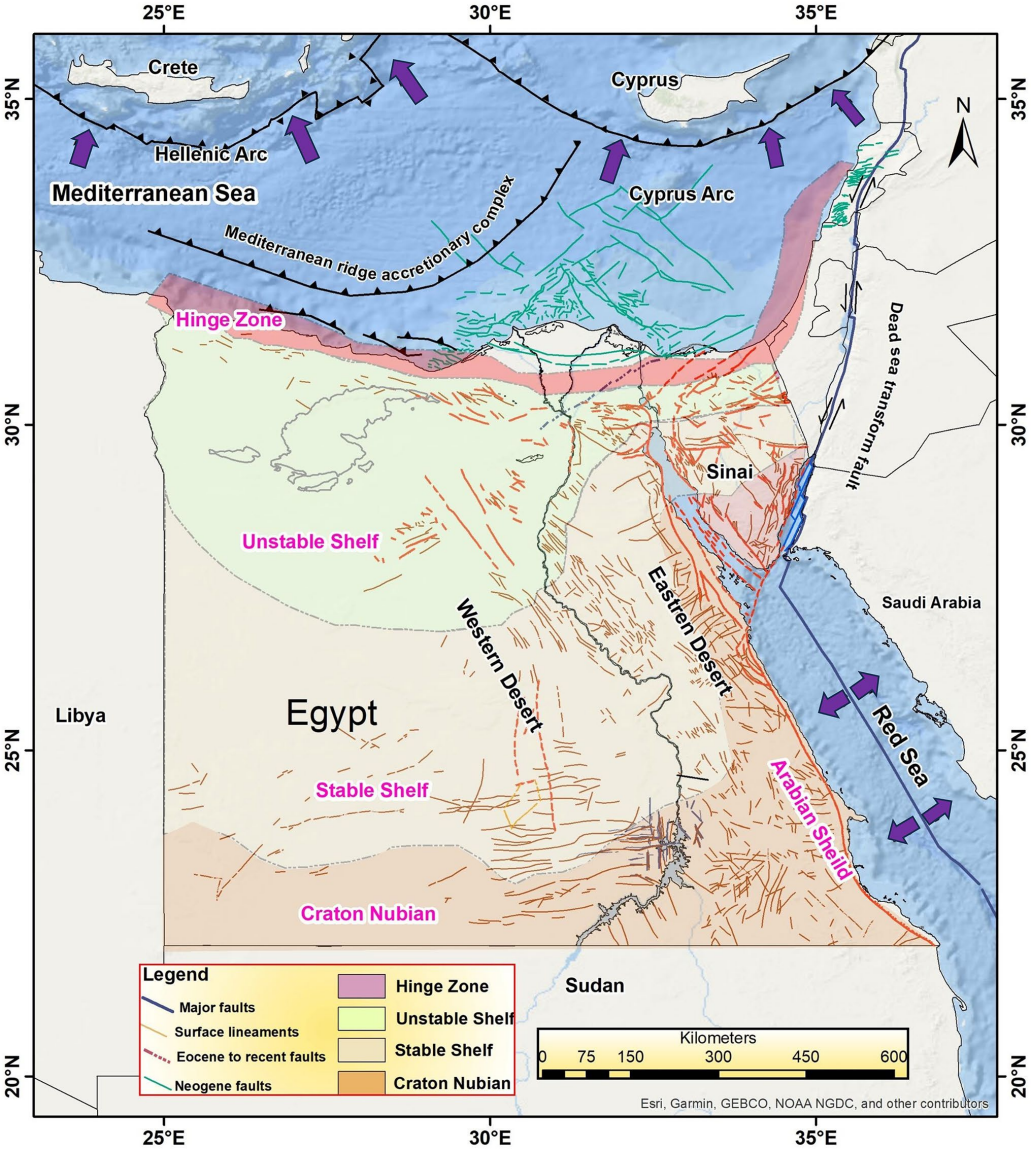
The main geological and tectonic features in and around Egypt. The geological features of Egypt are compiled from Meshref^20,21^. The major faults are compiled from McClusky et al.^22^; Abou Elenean and Hussein^23^. The surface lineaments, Eocene to recent faults, and Neogene faults are compiled from EGSMA^24^. Arrows show the direction of plate motion. This map was created by using ArcGIS 10.8.2 software and basemap produced by Ref.^16^.

The study area covers Egypt and the southeast part of the Mediterranean Sea; it extends from 21°N to 36° N and 22°E to 37°E. Egypt’s seismicity is characterized by both interplate and intraplate earthquakes, largely influenced by the movements between the African, Eurasian, and Arabian plates. Where it is surrounded by the Red Sea rift in the East and the Gulf of Aqaba - Dead Sea Transform Fault system in the northeast part. Subduction zone of the Mediterranean Sea is to the north of Egypt (Fig. 2). The most seismically active regions include the Eastern Mediterranean, the Gulf of Aqaba, the Gulf of Suez, and the northern Red Sea, where tectonic activity is most pronounced^19^.

The southern part of the eastern Mediterranean is a passive continental margin basin^25^. It is mainly controlled by NNW compressional stresses due to the tectonic movement between the African Plate and Eurasian Plate. The earthquake focal mechanism solutions in this area characterized either by reverse mechanisms along E-W to ENE-WSW trending faults or by normal mechanism along NNW trending faults^20^. The northern part of Egypt is characterized by normal faulting with strike-slip component. Where the study of Badway^20^ showed that all earthquake focal mechanism solutions in this zone has mainly pure normal mode of motion and oblique sense of dominant normal component associated with secondary component of right shear on E-W to NWN-SES and NW-SE striking planes which agree with the general strike direction of the exposed faults. In the southern Egypt, most of the earthquake activities are mainly in an E-W to ENE-WSW direction. The faulting mechanisms show two types of dislocation. The dominant one is strike-slip faulting demonstrated by a large number of solutions and the minor one is the normal dip slip with slight horizontal movement. Moreover, according to the literatures^21,26^. Egypt is divided into three main geological features; the Nubian-Arabian shield the stable shelf, the unstable shelf, and the Gulf of Suez-Red Sea Graben (Fig. 2).

### Dataset and processing

The dataset used in this study is collected from the available waveforms recorded by the ENSN, and other regional networks such as GEOFON, the Kandili Observatory, and the National Observatory of Athens Geodynamic Institute. We collect about 1670 events occurred in Egypt and neighbouring regions and took place from 2009 to 2019 with hypocentral depths less than 40 km.

**Fig. 3:**
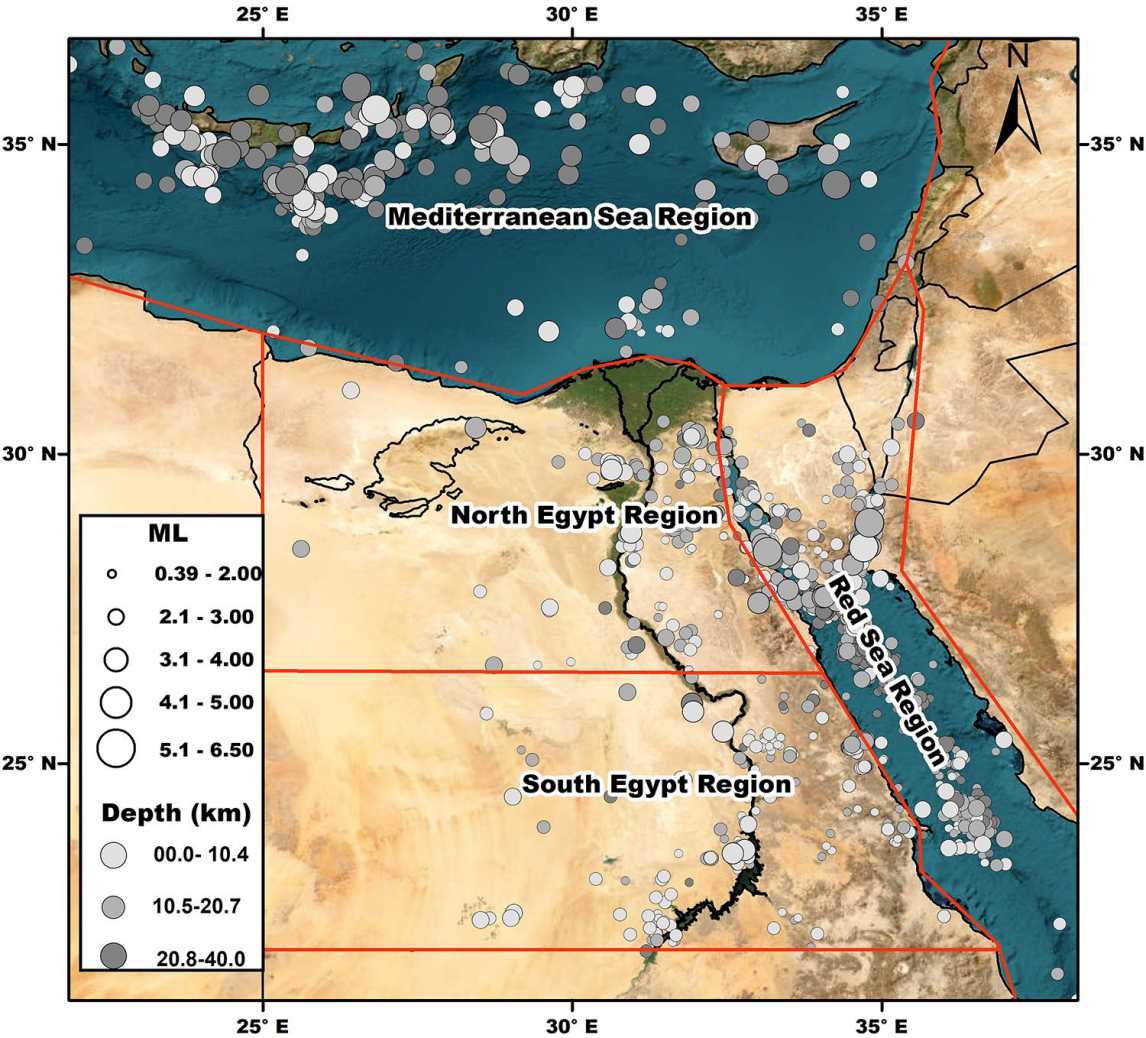
The spatial distribution of the collected earthquake dataset and the four sub-tectonic regions of Egypt. The circle size is proportional to the magnitude value (M_L_) and the circle colour express about the depth range (km) as illustrate in the legend of the map. The map was created by using ArcGIS 10.8.2 software and basemap produced by Ref.^16^.

Three main earthquake clusters can be distinguished: the first in the Eastern Mediterranean region, the second in the eastern part of Egypt, referred to in this study as the Red Sea region (including the Gulf of Suez and Gulf of Aqaba), and the third is related to intraplate earthquakes within the Egyptian territory. Due to Egypt’s heterogeneous geological setting, the third cluster is divided into two subregions: northern Egypt and southern Egypt. The selected earthquakes for this study were recorded by at least four seismic stations at hypocentral distances of less than 1000 km, with earthquake magnitudes ranging from 0.1 to 6.5 ML. The spatial distribution of the collected dataset and the four sub-tectonic regions of Egypt is illustrated in Fig. 3.

The dataset processing was conducted using the ATLAS earthquake analysis software (Nanometrics software). For each event, the process began with reviewing the picking of P- and S-waves from the observed seismograms and applying the appropriate crustal model for each region. Next, the instrument effect was removed to obtain true ground motion. The true ground motions were then simulated on a Wood Anderson (WA) torsion seismograph with a period of 0.8 s and a damping factor of 0.8. A gain of 2080 for the WA seismograph^27^ was assumed to anchor the new scale to the Richter scale. The WA simulation response was provided by poles and zero representation^28^ available in the earthquake analysis software.

**Fig. 4:**
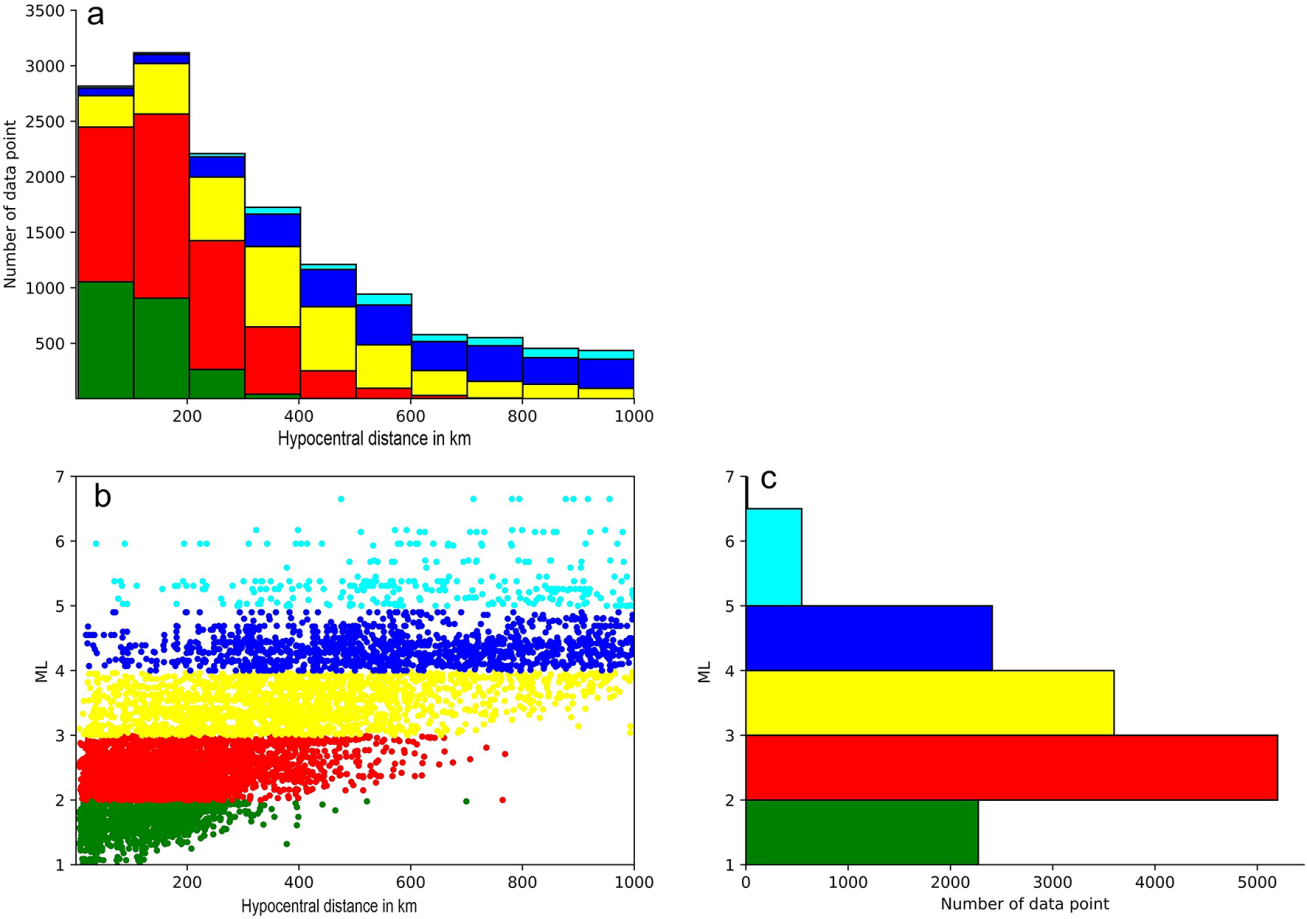
Distributions of magnitude and hypocentral observations. (**a**) the frequency distribution for the number of WA amplitude dataset and hypocentral distances. (**b**) the distribution of the Local magnitude ML and hypocentral distances. (**c**) The distribution of the Local magnitude ML and hypocentral distance.

Following the simulation of all seismic waves for each event, the maximum amplitude of the S-wave from the two horizontal components was measured and stored in a summary file. This file contained the maximum amplitude and epicentral distance for each horizontal component. The final dataset comprised 14,553 waveform amplitudes (Fig. 4).

It should be noted that significant saturation of local magnitude begins at 6.5. Consequently, events with a magnitude of 6.5 or less were selected to minimize errors related to the saturation of the ML scale^5,29^.

### Revelations on the attenuation attributes in Egypt

**Fig. 5:**
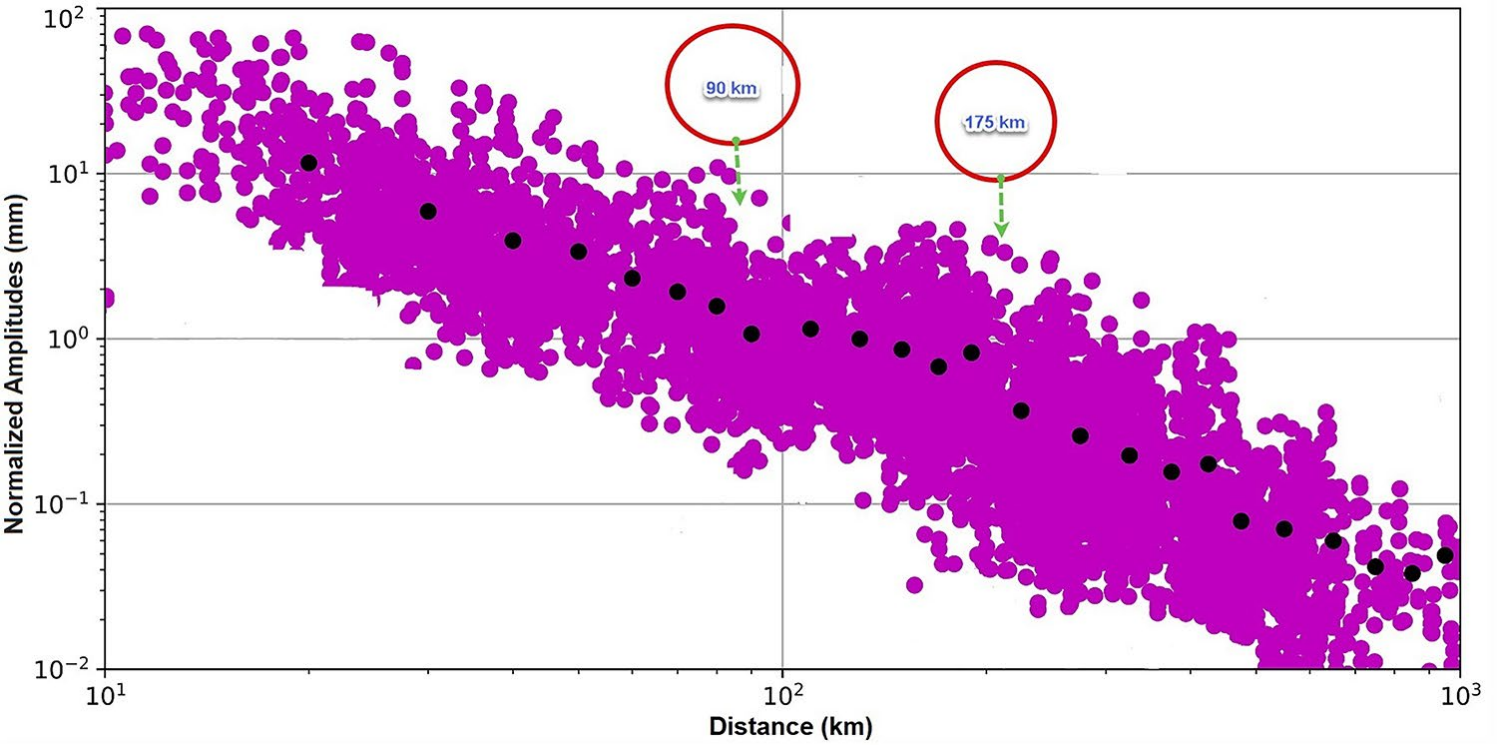
Normalized amplitudes (A_norm_) decay with distance for earthquakes, which have at least four amplitude measurements within a reference distance range. Black closed circles represent the logarithmic average of A_norm_ values determined for log-spaced distance bins for earthquakes.

The ground motion decay of amplitudes with distance is analysed to obtain information on regional attenuation effects. An extensive ground-motion data set encompassing the majority of Egypt is utilized as shown in Figure A-1 in the supplementary files. The WA amplitudes were scaled from various events to a typical level at a reference distance. For each event, the geometric mean of amplitudes is calculated at a given distance range, and then individual amplitudes are normalized at all distances by the mean value. This exercise is conducted for each event and reference distance bin, where these steps effectively eliminate differences in source effects, revealing the area’s attenuation features. It should be noted that normalized amplitudes always include site effects against the average site condition in each reference distance bin. A grid-search transition distances within 50 km ≤ R1 ≤ 150 km and 100 km ≤ R2 ≤ 300 km ranges with 10 km increments was estimated to calculate model coefficients for each R1–R2 combination via regression analysis. A logarithmic average of A_norm_ values was determined for log-spaced distance bins at every 10 km distance. This step could be a helpful way to get the pattern of noticeable change in amplitude decay with distance^13^. Overlapping R1 and R2 search windows also consider scenarios of bilinear geometrical spreading with no Moho bounce. As a matter of course, the average A_norm_ for all events achieves “one” at reference distance bins. Figure 5 shows a region where markedly amplitude decay slows between 90 and 175 km. This slow decay is emerging because of the Moho bounce effect of reflected and refracted phases joining direct waves, changing the intermediate distances of the attenuation scheme. The Moho bounce effect is used by Burger et al.^30^ to describe the interval of constant pseudo-velocity amplitudes between 100 and 200 km, caused by large-amplitude, postcritically reflected S waves from both the Moho and the interface at a depth of 30 km. It’s important to highlight that the Moho depth in Egypt fluctuates between 25 km in the northern part and 39 km in the southwestern region^31^.

Similar impacts were observed in North America between 70 and 150 km and in western Canada between 100 and 220 km^13^. The particular distance range and strength of the Moho bounce depend on crustal thickness, velocity structure, the dip angle of the Moho boundary, and regional anelastic attenuation^32,33^.

### Regression analysis

Based on the standard model of Hutton and Boore’s for the M_L_ scale relationship:

1 $$\:{M}_{L}=log\left(A\right)-log\left(A0\right)+S.$$

The distance correction term (− log A_0_) and the station correction term (S) have to be identified. The − log A_0_ is described in the frame of the trilinear geometrical spreading model. Which is first proposed by Refs.^32,34^ to assess the geometrical spreading and anelastic attenuation behaviours by fixing the coefficients of the geometrical spreading to the theoretical values and searched for the hinge positions of a tri-linear model, viz., separate values in three distances segments.

Recent studies for estimation the M_L_ Scale^13,35^ used the geometrical spreading model to account the observed attenuation effect and define the distance correction term (− log A_0_) as:

2 $$- {\text{ log A}}0\,=\,{\text{GS}}\,+\,{\text{g}}\left( {{\text{R }}-{\text{ 1}}00} \right)\,+\,{\text{3}},$$

Where GS represents the geometric spreading and γ is the anelastic scattering effects for specific region. R is hypocentral distance (km). If Eq. (2) is constrained to Ao = 0.001 mm and *R* = 100 km as in the case of zero-magnitude earthquake definition in Richter^6^ and in this case both GS and γ are defined as Z and the geometric spreading correction is given as:

3 $${\text{GS}}\,=\,{\text{Z}}\left( {\text{R}} \right){\text{ }}-{\text{ Z}}\left( {{\text{R}}\,=\,{\text{1}}00{\text{ km}}} \right),$$

According to Refs.^13,35^ Z in Eq. (3) is a hinged-trilinear function of a distance and present the decay of the decay o WA amplitudes as follows:

4 $$\begin{gathered} {\text{Z}}\left( {\text{R}} \right)\,=\,\left\{ {\begin{array}{*{20}{c}} {{\text{b1 log}}\left( {\text{R}} \right)\,}&{{\text{R}}\, \leqslant \,{\text{R1}}} \\ {{\text{b1 log}}\left( {{\text{R1}}} \right)\,+\,{\text{b2 log }}\left( {{\text{R/R1}}} \right)}&{{\text{R1}}\,<\,{\text{R}}\, \leqslant \,{\text{R2}}} \\ {{\text{b1 log}}\left( {\text{R}} \right)\,+\,{\text{b2 log }}\left( {{\text{R2}}/{\text{R1}}} \right)\,+\,{\text{b3 log }}\left( {{\text{R/R2}}} \right)}&{{\text{R}}\,>\,{\text{R2}}} \end{array}\,} \right., \hfill \\ {\text{ }} \hfill \\ \end{gathered}$$

Parameters b1, b2, and b3 are geometrical spreading coefficients for the distance ranges separated by hinge points at R1 and R2 km which equivalent to the attenuation attributes in Egypt at 90 km and 175 km, respectively.

The observed WA amplitudes are regressed based on the pervious equations (Eq. 1 to 4) to establish the coefficients of the regional distance correction model (b1, b2, b3, and γ).

The station correction term (S) is the value to be added to the single station magnitude to compensate for the difference between the station magnitude and the WA magnitude. It is derived under the constraint that the station corrections sum is close to zero when averaged over all stations.

## Results and discussion

The regional attenuation analysis for the decay of 14,453 normalized WA amplitudes reveals the presence of two transition distance at 90 km and 175 km producing three segments of attenuation and according to Atkinson and Mereu^34^ each segment is corresponding to attenuation of seismic phase as follows:

i. Corresponding to attenuation of direct seismic wave at distance from 0 to 90 km.
ii. Corresponding to direct waves joined by the reflections from mid-crustal interfaces and the moho discontinuity at distance from 90 to 175 km.
iii. Corresponding to attenuation of multiply reflected and refracted S waves at distances more than 175 km.

Due to the heterogeneity in geological condition entire Egyptian territory, Egypt has been divided into four sub-tectonic regions: Southern Egypt, North Egypt, the Red Sea region, and the Mediterranean Sea region, where the least square linear regression analysis is imposed separately on each sub-tectonic zone.

**Fig. 6:**
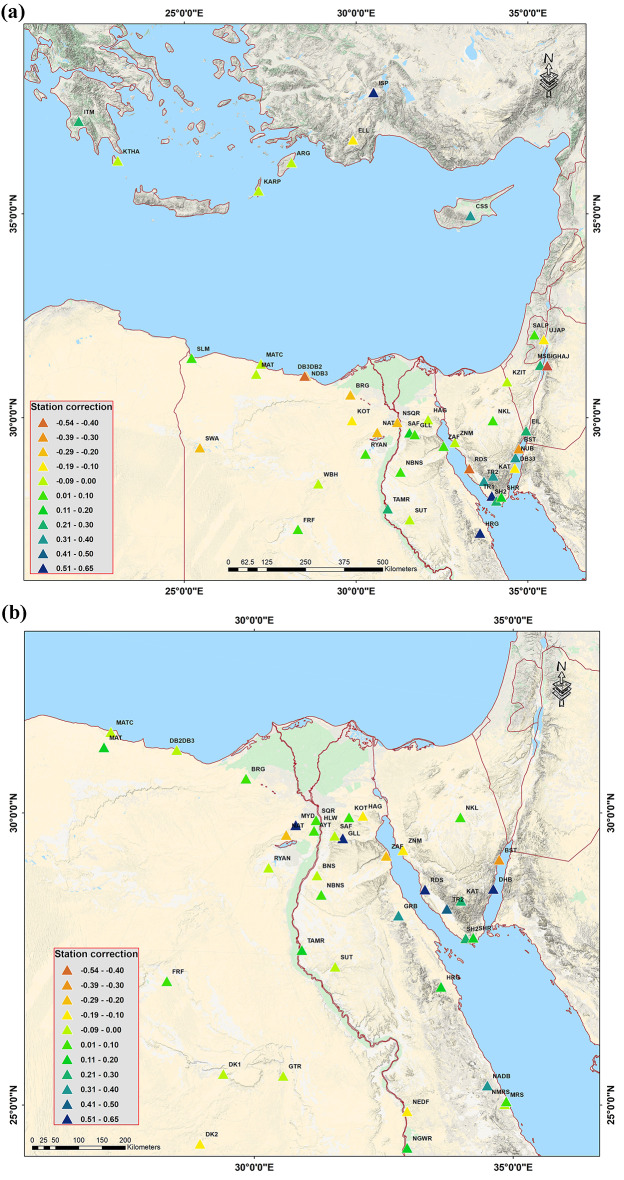

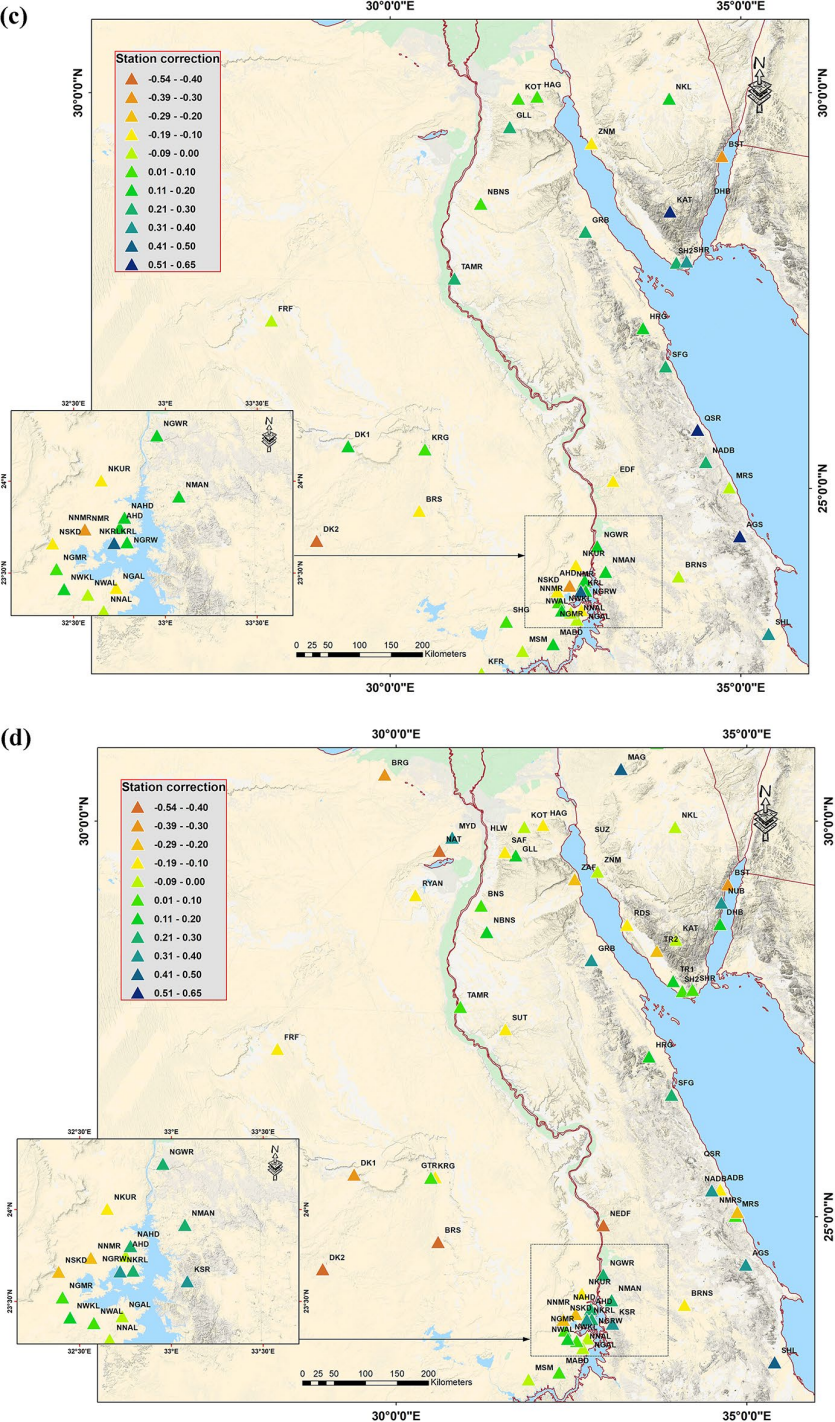
Station corrections, computed relative to the average of all stations from local and regional networks, are shown for 100 stations. (**a**) the Mediterranean Sea., (**b**) Northern Egypt, (**c**) Southern Egypt, and (**d**) the Red Sea. (refer to the appendices (1–4) For more details. These maps were created by sing ArcGIS 10.7 softwarae and basemap produced by Ref.^37^.

The coefficients of the best-fitting − log A_0_ model are listed in Table 1 for each region. Station corrections computed relative to the average of all stations from local and regional networks are shown for 100 stations in Fig. 6, (refers to Appendix for more details). The 100 site corrections (obtained from the inversion) are primarily between (− 0.7 and 0.7). The corrections are positive in Egypt’s Red Sea coast, which consists of igneous and metamorphic rocks, and negative in most other locations in Egypt, where sedimentary rock is predominant. A station with a positive correction will yield a smaller ground motion value than a station with a negative correction for any seismic event before the station corrections are applied. These may result from highly localized effects on the site, such as near-surface geology and the degree of coupling between the seismometer and the soil.

**Table 1:** Coefficients of distance correction model (− log A0) for M_L_ scale in Egyptian sub-regions (with refers to Eqs. 4 and 5).

Sub-region	b1 (std)	b2 (std)	b3 (std)	γ (std)
Red sea	1.043587 (± 0.00664)	1.659729 (± 0.0372)	2.1249367 (± 0.008299)	− 0.0003992 (± 3e-05)
Mediterranean sea	0.970793 (± 0.040632)	2.834864 (± 0.49203)	0.0819252 (± 0.04227)	0.00129 (± 9.1e-5)
North Egypt	1.2805915 (± 0.03563)	1.31100518 (± 0.2002)	1.990877 (± 0.04001)	− 0.0003439 (± 0.000143)
South Egypt	1.4223325 (± 0.02260)	1.37860939 (± 0.15325)	3.136902 (± 0.04329)	− 0.0014125 (± 0.00017)

**Fig. 7:**
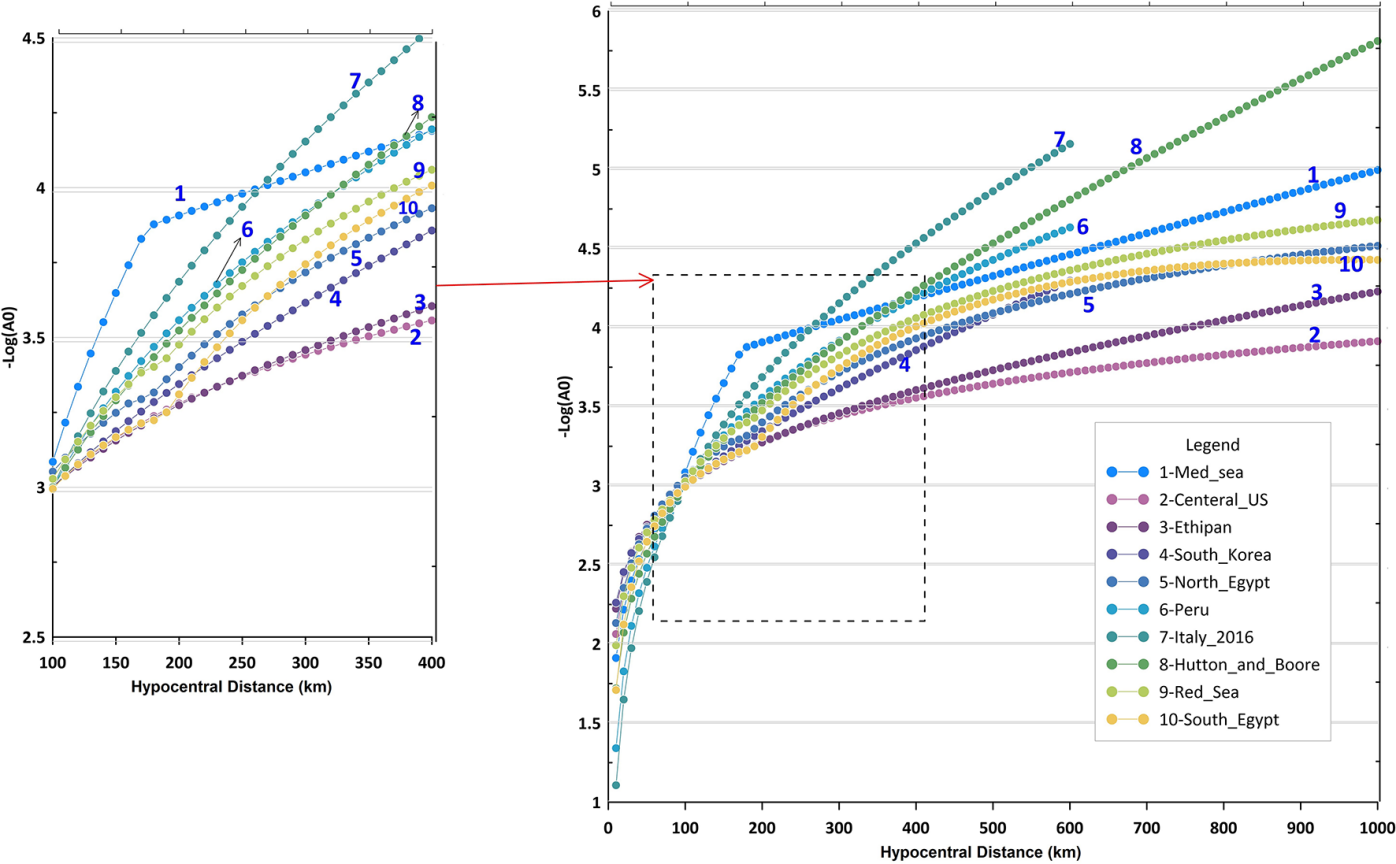
Comparison of obtained attenuation curves (South Egypt, North Egypt, Mediterranean Sea, and Red Sea) with different local magnitude scales for southern California (Hutton and Boore 1987), Ethiopian Plateau (Brazier et al. 2008), Central US (Miao and Langston 2007), Italy (Bona 2016), Peru (Condori et al. 2017), and Korea (Sheen et al. 2018). The inset graph zoomed-in on the comparison at a distance range 200–500 km.

For the sake of comparison, the attenuation curves for southern California^2^, Ethiopian Plateau^34^, Central United States^36^, Italy^11^, Peru^37^ and Korea^38^, with ENSN (Northern Egypt, South Egypt, Red Sea and, the Eastern Mediterranean) are plotted in Fig. 7. In general, the variation between all attenuation curves is minimal at short distances (less than 100 km). Beyond that, the attenuation curves from this study are intermediate between those from the central United States and southern California for the entire distance range (up to 1000 km). For distances less than 600 km, it is noted that the South Egypt attenuation curve closely aligns with the South Korea curve.

The Mediterranean attenuation curve shows a slight deviation from all other curves at distances between 100 and 300 km. The tectonic state of the eastern Mediterranean is complex, with most seismic activity concentrated in the Hellenic Arc. This activity primarily occurs offshore, with significant events taking place between the deepest part of the trench and the islands. Additionally, the deviation of the Mediterranean curve is influenced by the limited number of seismic stations. The challenge of installing seismic stations in the sea at these distances (100–300 km) could ultimately impact the results of the least squares inversion process.

**Fig. 8:**
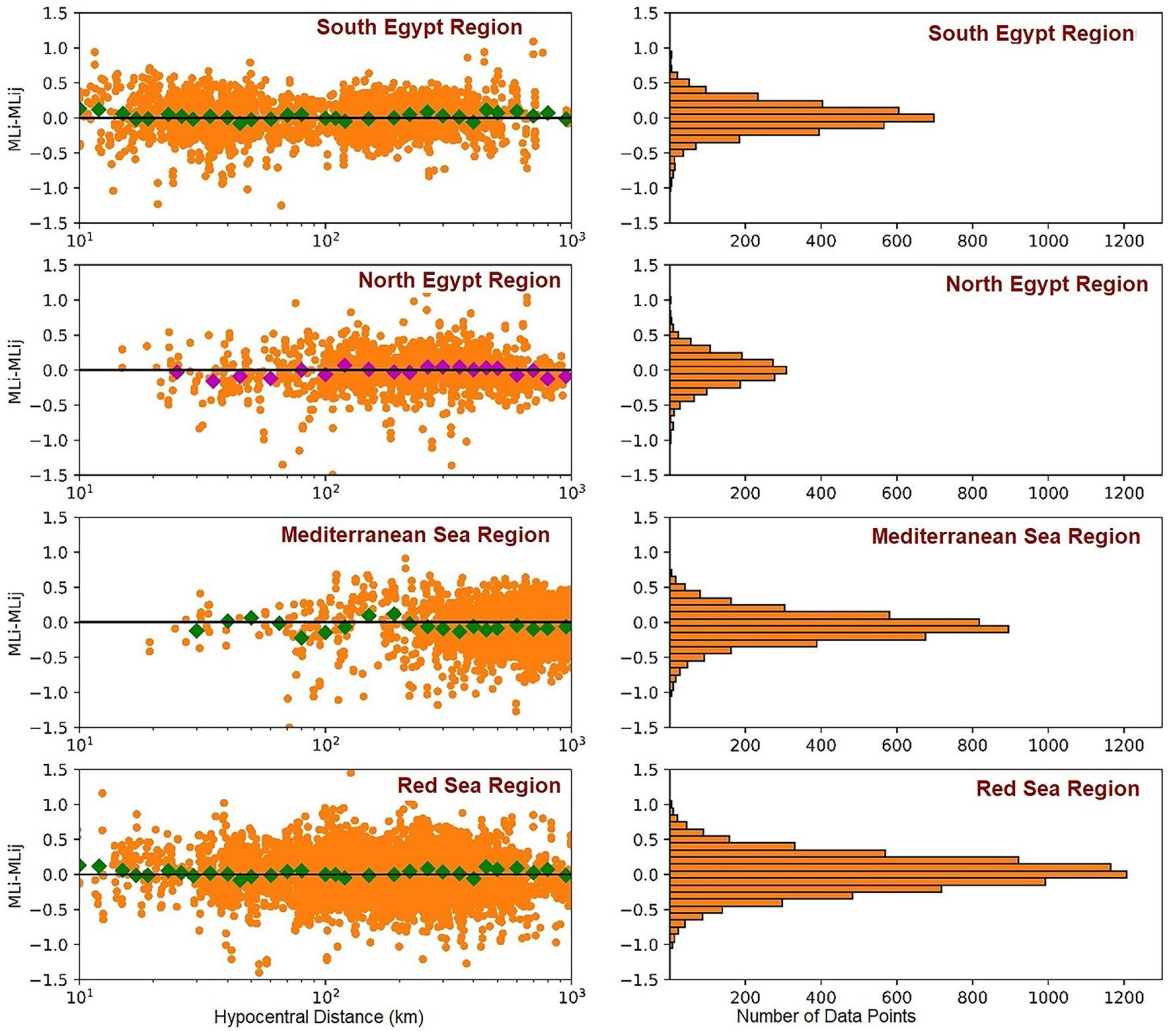
Residuals variation of M_L_ relation as a distance function. Large colored squares represent a residual average for earthquakes.

**Fig. 9:**
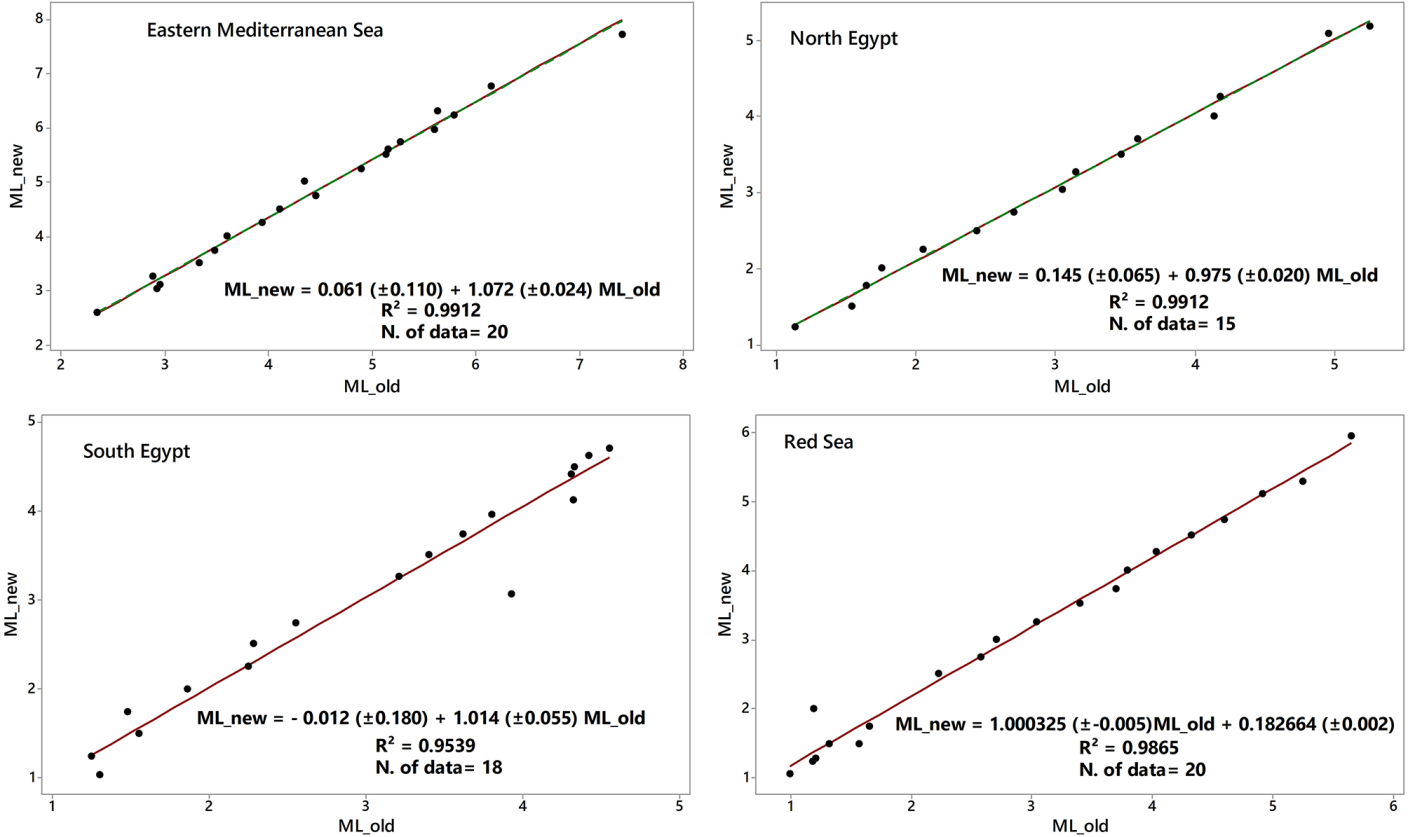
Plots of orthogonal regression relations between ML obtained by the derived scales the current study (ML_new) and the ML applied by ENSN (ML_old) for each dataset.

The variation of magnitude residuals to the distance for all events in the compiled datasets is shown in Fig. 8(left Panels). The magnitude residuals are defined as M_Lij_ – M_Li_, in which M_Lij_ is the magnitude estimate for station j of event i based on the ENSN models, and M_Li_ is the mean magnitude of the event. The right panels of Fig. 9 show that mean residuals reach values close to zero, irrespective of the distance up to 1000 km. These showed no particular trend regarding either distance or logarithmic distance and imply that the results represent the attenuation characteristics in and around Egypt. Station corrections help to minimize residue deviations. There is a small residual gap from zero for distances less than 200 km for the Mediterranean region. As mentioned, this could arise from the lack of seismic stations. Overall, the derived relation well captures the attenuation characteristics in ENSN over a vast distance range, providing a reduced scatter of M_L_ estimations across different stations due to empirical station corrections (S).

The retrieved Local Magnitude Scales for Egypt are presented in Table 2.

**Table 2:** The derived local magnitude scales ($\:{M}_{L})$ for Egypt (A the measured amplitude (mm) and R is the hypocentral distance (km)).

Sub-region	$\:{M}_{L}$ scale	*R* (km)
Red sea	log (A) + 1.043587165694*log(R) -0.0003992*(R) + 0.9527	< 90
	log (A) + 1.6597293119377*log(R) -0.0003992*(R) − 0.251	90–175
	log (A) + 2.12493673826099*log(R) -0.0003992*(R) − 1.29	> 175
Mediterranean sea	log (A) + 0.97079375*log(R) + 0.00129*(R) + 0.929	< 90
	log (A) + 2.8348645548*log(R) + 0.00129*(R) -2.71343	90–175
	log(A) + 0.08192525*log(R) + 0.00129*(R) + 3.461	> 175
North Egypt	log (A) + 1.28059159180543*log(R) -0.0003439*(R) + 0.4732	< 90
	log (A) + 1.31100518479357*log(R) -0.0003439*(R) + 0.413772	90–175
	log (A) + 1.99087770179433*log(R) -0.0003439*(R) -1.11121	> 175
South Egypt	log (A) + 1.42233250326395*log(R) -0.0014125*(R) + 0.301	< 90
	log (A) + 1.37860939573344*log(R) -0.0014125*(R) + 0.379	90–175
	log (A) + 3.1369021102549*log(R) -0.0014125*(R) -3.57	> 175

Plausibility check for the coefficients of the attenuation for the local magnitude scales in Egypt is presented by analyzing the orthogonal regression for the event magnitudes determined based on the new M_L_ scales and the old M_L_ scale for the four regions (Fig. 8). The orthogonal regression is presented by considering the error variance ratio equal to unity^39^. The Orthogonal regression relations between M_L_ obtained by the derived scales of the current study and the M_L_ applied by ENSN for each dataset are presented in Table 3.

**Table 3:** Orthogonal regression relations between ML obtained by the derived scales the current study (ML_new) and the ML applied by ENSN (ML_old) for each dataset.

Data set	Coefficients of relationship	*R*²
Red sea	ML_new = 0.183 (± 0.046) + 1.000 (± 0.010) ML_old	0.9865
North Egypt	ML_new = 0.145 (± 0.065) + 0.975 (± 0.020) ML_old	0.9945
Mediterranean sea	ML_new = 0.061 (± 0.110) + 1.072 (0.024) ML_old	0.9912
South Egypt	ML_new = − 0.012 (± 0.180) + 1.014 (± 0.055) ML_old	0.9539

It is noteworthy that, the first attempt for applying the trilinear geometrical scattering model and singular value decomposition to retrieve the local magnitude scale in Egypt is done recently by Abdullah et al.^18^. However, their study was limited to the southern part of Egypt. Our study incorporates the southern part of Egypt and yielded similar results.

To enrich our discussion, a question is raised about how far the new magnitude scales could improve the frequency-magnitude distribution for each sub region. The Gutenberg-Richter distribution is calculated for each zone using the old magnitudes and the new ones obtained from the retrieved scales. As shown in Figures (A-2) in supplementary documents, the differences aren’t large. These differences may be due to: (i) the completeness of the dataset considered (lower magnitudes) and (ii) the short observation interval (higher magnitudes), along with the saturation of the M_L_ scale.

## Conclusions

New local magnitude scales have been developed for Egypt, covering southern Egypt, northern Egypt, the Red Sea region, and the Mediterranean Sea region. Approximately 14,453 maximum amplitudes measured on Wood-Anderson seismograms from 1,670 local and regional earthquakes were utilized. The magnitude range under investigation is 1.0 to 6.5 in and around Egypt from 2009 to 2019. Amplitude decay was checked against hypocentral distance to reveal attenuation behavior, using a new distance normalization of 0.001 mm displacement recorded at 100 km for a magnitude of 3.0. A significant Moho bounce effect was detected at 90 and 175 km hypocentral distances, explaining the observed attenuation attributes.

A trilinear regression model was applied to the observed amplitudes dataset to model regional attenuation parameters for selected sub-areas. Site corrections were also determined for each station’s subregions, allowing consistent ML estimates regardless of site conditions.

Based on robust modeling of the region’s attenuation effects, magnitude relationships derived from ENSN ensure accurate M_L_ estimates over a wide range of distances (up to 1000 km). Most residual changes between the estimated M_L_ results and a median magnitude appear near zero at any distance up to 1000 km. Lastly, the authors suggest that a study of varying magnitude in the eastern Mediterranean is necessary in the future due to its tectonic complexity.

## Electronic supplementary material

Below is the link to the electronic supplementary material.

Supplementary Material 1

## Data Availability

The datasets used and analysed during the current study are available from the corresponding author on reasonable request.

## Declarations

### Information and resources

The Egyptian National Seismic Network (ENSN) recorded waveform data used in this study. The study also included data from regional networks GEOFON Data Centre (GE) and Kandili Observatory Digital Broadband Seismic Network (KO). Event detections, phase arrivals, event locations, and amplitude picks are manually reviewed. Regression analyses are performed using python 3.7 (https://docs.anaconda.com/anaconda/packages/py3.7_win-64/*)*, and maps are produced by GMT 5.4 (http://www.soest.hawaii.edu/gmt/*)* and ArcMap 10.8.2(*arcgis.com*).
