# Publisher Correction: New local magnitude scales for Egypt

**DOI:** 10.1038/s41598-025-91354-9

**Published:** 2025-02-25

**Authors:** Sherif M. Elhady, Mohamed Ezzelarab, M. Sami Soliman, Hussein S. Abdullah, Iman F. Abu El Nader, Ashraf Adly, Tharwat H. Abd-Elhafeez

**Affiliations:** 1 Egyptian National Seismic Network (ENSN), Department of Seismology, National Research Institute of Astronomy and Geophysics, Cairo, Egypt; 2 Geology Department, Faculty of Science, Al-Azhar University, Cairo, Egypt

Correction to: *Scientific Reports* 10.1038/s41598-024-80995-x, published online 23 December 2024

The original version of this Article contained errors in the Figure legends of Figure 8 and Figure 9. The legends of these Figures were inadvertently switched.

**The legend of Figure 8:**

“Plots of orthogonal regression relations between ML obtained by the derived scales of the current study (ML_new) and the ML applied by ENSN (ML_old) for each dataset.”

now reads:

“Residuals variation of M_L_ relation as a distance function. Large colored squares represent a residual average for earthquakes.”

**The legend of Figure 9:**

“Residuals variation of M_L_ relation as a distance function. Large colored squares represent a residual average for earthquakes.”

now reads:

“Plots of orthogonal regression relations between ML obtained by the derived scales of the current study (ML_new) and the ML applied by ENSN (ML_old) for each dataset.”

The original Article has been corrected.

