# Supplementary material for: New local magnitude scales for Egypt
Source: Sci Rep. 2024 Dec 23;14:30589. doi: 10.1038/s41598-024-80995-x (PMC11666577; doi:10.1038/s41598-024-80995-x)
Supplement: Supplementary file 1 — Supplementary Material 1 [file 41598_2024_80995_MOESM1_ESM.docx]

**Supplementary documents**

### A1: Regional coverage of the surface projection of ray path


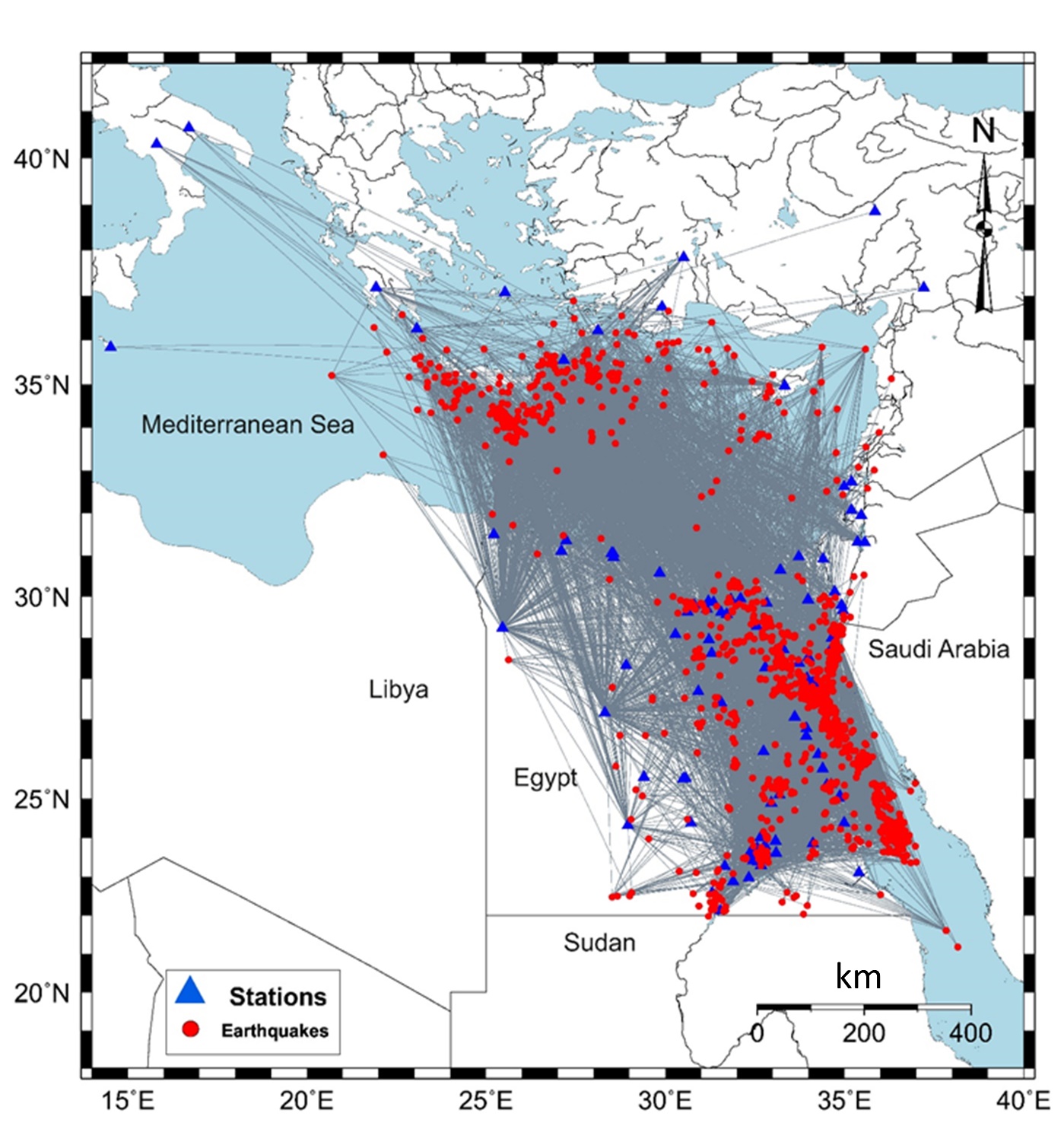


Figure A-1: Regional coverage of the surface projection of ray paths for study events. This map was created by Generic Mapping Tools (GMT) version 5.4.

### A2: Station corrections tables:

| **Table (A-1)** Mediterranean Sea (Station Correction) | | | | | | |
| --- | --- | --- | --- | --- | --- | --- |
| **Network** | **Network Code** | **St. code** | **Sensor type** | **Longitude (E⸰)** | **Latitude (N°)** | **Station Correction (S)** |
| [GEOFON Program, GFZ Potsdam, Germany](https://geofon.gfz-potsdam.de/waveform/archive/network.php?ncode=GE) | GE | MARC | Trillium-Compact-120 | 15.8063 | 40.297 | 0.651 |
|  | GE | ISP | STS-1 | 30.5093 | 37.843 | 0.511 |
|  | GE | CSS | STS-2 | 33.331 | 34.961 | 0.326 |
|  | GE | EIL | STS-2 | 34.9512 | 29.67 | 0.28 |
|  | GE | MSBI | Trillium-240 | 35.358 | 31.313 | 0.254 |
|  | GE | SALP | Trillium-240 | 35.1914 | 32.069 | 0.0199 |
|  | GE | WBH | Trillium 120 | 28.9038 | 28.3208 | -0.00227 |
|  | GE | MATE | STS-2 | 16.7044 | 40.649 | -0.009 |
|  | GE | KARP | STS-2 | 27.1611 | 35.5471 | -0.0558 |
|  | GE | KTHA | STS-2 | 23.0621 | 36.2566 | -0.0633 |
|  | GE | UJAP | Trillium-240 | 35.4644 | 31.952 | -0.146 |
|  | GE | GHAJ | Trillium-240 | 35.5665 | 31.3034 | -0.545 |
| Helwan/ Egyptian national seismological network (ENSN) | HL | HRG | Trillium-40 | 33.6081 | 27.0517 | 0.582 |
|  | HL | NNAL | Trillium-40 | 32.6647 | 23.2931 | -0.0731 |
|  | HL | NWAL | Trillium-40 | 32.5779 | 23.3829 | 0.182 |
|  | HL | NWKL | Trillium-40 | 32.44888 | 23.41309 | 0.114 |
|  | HL | NGAL | Trillium-40 | 32.7324 | 23.4192 | 0.0779 |
|  | HL | NGMR | Trillium-40 | 32.40756 | 23.52169 | 0.215 |
|  | HL | NSKD | Trillium-40 | 32.386 | 23.661 | -0.142 |
|  | HL | NGRW | Trillium-40 | 32.7912 | 23.6684 | 0.454 |
|  | HL | NNMR | Trillium-40 | 32.5621 | 23.7379 | -0.0392 |
|  | HL | NAHD | Trillium-40 | 32.778 | 23.8022 | 0.477 |
|  | HL | BRNS | Trillium-240 | 34.1143 | 23.8559 | 0.123 |
|  | HL | NMAN | Trillium-40 | 33.0737 | 23.9169 | 0.369 |
|  | HL | NKUR | Trillium-40 | 32.6514 | 24.0042 | 0.0399 |
|  | HL | MRS | Trillium 120 | 34.8387 | 25.0131 | 0.532 |
|  | HL | FRF | Trillium-240 | 28.3105 | 27.1484 | 0.00697 |
|  | HL | SUT | T120C | 31.5626 | 27.3967 | -0.079 |
|  | HL | TAMR | Trillium-240 | 30.9175 | 27.6821 | 0.244 |
|  | HL | SH2 | Trillium-240 | 34.0833 | 27.8817 | 0.202 |
|  | HL | SHR | Trillium 120 | 34.2301 | 27.9847 | 0.184 |
|  | HL | TR1 | Trillium-40 | 33.9521 | 28.0068 | 0.509 |
|  | HL | TR2 | Trillium Compact-120 | 33.7227 | 28.3853 | 0.322 |
|  | HL | KAT | Trillium-40 | 33.9928 | 28.5229 | 0.315 |
|  | HL | NBNS | Trillium-40 | 31.2945 | 28.6226 | 0.0939 |
|  | HL | RDS | SS1 | 33.2975 | 28.712 | -0.447 |
|  | HL | DB3 | Trillium-40 | 34.6188 | 28.7221 | -0.174 |
|  | HL | NUB | SS1 | 34.6396 | 28.9893 | 0.351 |
|  | HL | RYAN | Trillium 120 | 30.27754 | 29.08251 | 0.0392 |
|  | HL | BST | Trillium-40 | 34.7327 | 29.2166 | -0.39 |
|  | HL | SWA | Trillium-240 | 25.4556 | 29.2432 | -0.236 |
|  | HL | ZAF | Trillium 120 | 32.5487 | 29.2819 | 0.00451 |
|  | HL | ZNM | SS1 | 32.8752 | 29.3761 | -0.0873 |
|  | HL | GLL | SS1 | 31.7081 | 29.5772 | 0.0182 |
|  | HL | SAF | Trillium 120 | 31.5538 | 29.6187 | 0.11 |
|  | HL | NAT | Trillium 120 | 30.6172 | 29.6329 | -0.215 |
|  | HL | NSQR | Trillium 120 | 31.2015 | 29.8832 | -0.24 |
|  | HL | KOT | STS-2 | 29.8832 | 29.9276 | -0.102 |
|  | HL | NKL | Trillium-240 | 33.9804 | 29.9293 | 0.0105 |
|  | HL | HAG | SS1 | 32.099 | 29.953 | -0.0371 |
|  | HL | BRG | SS1 | 29.8393 | 30.5743 | -0.24 |
|  | HL | NDB3 | Trillium-40 | 28.5482 | 31.0378 | -0.238 |
|  | HL | DB3 | Trillium-40 | 28.50385 | 31.04697 | -0.0254 |
|  | HL | DB2 | Trillium-40 | 28.5039 | 31.047 | -0.418 |
|  | HL | MAT | Trillium-40 | 27.0964 | 31.094 | -0.0386 |
|  | HL | MATC | L4C | 27.2305 | 31.3457 | -0.0505 |
|  | HL | SLM | Trillium-240 | 25.2123 | 31.4916 | 0.0397 |
| [National Observatory of Athens Seismic Network](https://www.fdsn.org/networks/detail/HL/) | HL | ARG | Le3D/20 | 28.12122 | 36.21356 | -0.0354 |
|  | HL | ITM | Le3D/20 | 21.9252 | 37.1787 | 0.228 |
| [Kandilli Observatory And Earthquake Research Institute (KOERI)](https://www.fdsn.org/networks/detail/KO/) | KO | GAZ | Guralp CMG-3T | 37.2097 | 37.172 | 0.0868 |
|  | KO | ELL | Güralp 3T | 29.9085 | 36.7483 | -0.121 |

| **Table (A-2)** Red Sea (Station Correction) | | | | | | |
| --- | --- | --- | --- | --- | --- | --- |
| **Network** | **Network Code** | **St. code** | **Sensor type** | **Longitude (E⸰)** | **Latitude (N°)** | **Station Correction (S)** |
| Helwan/ Egyptian national seismological network (ENSN) | HL | MSM | SS1 | 31.889 | 22.8814 | -0.0903 |
|  | HL | MABD | Trillium-240 | 32.3258 | 22.9726 | 0.00425 |
|  | HL | SHL | SS1 | 35.3999 | 23.1067 | 0.413 |
|  | HL | NNAL | Trillium-40 | 32.6647 | 23.2931 | -0.0826 |
|  | HL | NWAL | Trillium-40 | 32.5779 | 23.3829 | 0.0104 |
|  | HL | NWKL | Trillium-40 | 32.44888 | 23.41309 | 0.105 |
|  | HL | NGAL | Trillium-40 | 32.7324 | 23.4192 | -0.0497 |
|  | HL | NGMR | Trillium-40 | 32.40756 | 23.52169 | 0.0271 |
|  | HL | KSR | Trillium-40 | 33.0872 | 23.6105 | 0.395 |
|  | HL | NSKD | Trillium-40 | 32.386 | 23.661 | -0.297 |
|  | HL | NKRL | Trillium-40 | 32.7211 | 23.6634 | 0.31 |
|  | HL | NGRW | Trillium-40 | 32.7912 | 23.6684 | 0.234 |
|  | HL | NNMR | Trillium-40 | 32.5621 | 23.7379 | -0.205 |
|  | HL | AHD | Trillium-40 | 32.751 | 23.7474 | -0.0749 |
|  | HL | NAHD | Trillium-40 | 32.778 | 23.8022 | 0.27 |
|  | HL | BRNS | Trillium-240 | 34.1143 | 23.8559 | -0.123 |
|  | HL | NMAN | Trillium-40 | 33.0737 | 23.9169 | 0.259 |
|  | HL | NKUR | Trillium-40 | 32.6514 | 24.0042 | -0.13 |
|  | HL | NGWR | Trillium-40 | 32.9535 | 24.2495 | 0.286 |
|  | HL | DK2 | Trillium 120 | 28.9546 | 24.3195 | -0.474 |
|  | HL | AGS | Trillium 120 | 34.9866 | 24.3794 | 0.38 |
|  | HL | NEDF | Trillium 120 | 32.9564 | 24.8903 | -0.429 |
|  | HL | MRS | Trillium 120 | 34.8387 | 25.0131 | 0.011 |
|  | HL | NMRS | Trillium-40 | 34.868 | 25.063 | -0.256 |
|  | HL | NADB | Trillium-240 | 34.5021 | 25.3405 | 0.36 |
|  | HL | ADB | Trillium-240 | 34.6238 | 25.351 | -0.167 |
|  | HL | KRG | SS1 | 30.4985 | 25.5032 | 0.0844 |
|  | HL | GTR | SS1 | 30.5595 | 25.5096 | -0.113 |
|  | HL | DK1 | Trillium 120 | 29.4028 | 25.5432 | -0.357 |
|  | HL | QSR | Trillium 120 | 34.3871 | 25.7554 | 0.664 |
|  | HL | SFG | Trillium 120 | 33.9292 | 26.5675 | 0.202 |
|  | HL | HRG | Trillium 120 | 33.6081 | 27.0517 | 0.183 |
|  | HL | FRF | Trillium-240 | 28.3105 | 27.1484 | -0.191 |
|  | HL | SUT | T 120C | 31.5626 | 27.3967 | -0.128 |
|  | HL | TAMR | Trillium-240 | 30.9175 | 27.6821 | 0.027 |
|  | HL | SH2 | Trillium-240 | 34.0833 | 27.8817 | 0.000964 |
|  | HL | SHR | Trillium 120 | 34.2301 | 27.8947 | 0.0913 |
|  | HL | TR1 | Trillium-40 | 33.9521 | 28.0068 | 0.186 |
|  | HL | GRB | SS1 | 32.7859 | 28.2705 | 0.314 |
|  | HL | TR2 | Trillium Compact | 33.7227 | 28.3853 | -0.255 |
|  | HL | KAT | Trillium-40 | 33.9928 | 28.5229 | -0.0687 |
|  | HL | NBNS | Trillium-40 | 31.2945 | 28.6226 | 0.164 |
|  | HL | RDS | SS1 | 33.2975 | 28.712 | -0.125 |
|  | HL | DHB | L4C | 34.6188 | 28.7221 | 0.189 |
|  | HL | BNS | Trillium 120 | 31.2126 | 28.9517 | 0.057 |
|  | HL | NUB | SS1 | 34.6396 | 28.9893 | 0.363 |
|  | HL | RYAN | Trillium 120 | 30.27754 | 29.08251 | -0.127 |
|  | HL | BST | Trillium-40 | 34.7327 | 29.2166 | -0.356 |
|  | HL | SWA | Trillium-240 | 25.4556 | 29.2432 | -0.159 |
|  | HL | ZAF | Trillium 120 | 32.5487 | 29.2819 | -0.283 |
|  | HL | ZNM | SS1 | 32.8752 | 29.3761 | -0.0717 |
|  | HL | GLL | SS1 | 31.7081 | 29.5772 | 0.197 |
|  | HL | SAF | Trillium 120 | 31.5538 | 29.6187 | -0.128 |
|  | HL | NAT | Trillium 120 | 30.6172 | 29.6329 | -0.441 |
|  | HL | MYD | L4C | 30.8009 | 29.7958 | 0.305 |
|  | HL | SUZ | SS1 | 32.8322 | 29.8406 | -0.681 |
|  | HL | HLW | SS1 | 31.3432 | 29.8585 | 0.737 |
|  | HL | KOT | STS-2 | 31.8292 | 29.9276 | -0.07 |
|  | HL | NKL | Trillium-240 | 33.9804 | 29.9293 | -0.0778 |
|  | HL | HAG | SS1 | 32.099 | 29.953 | -0.187 |
|  | HL | BRG | SS1 | 29.8393 | 30.5743 | -0.358 |
|  | HL | MAG | SS1 | 33.2082 | 30.643 | 0.424 |
|  | HL | RSH | SS1 | 33.7219 | 30.9601 | 0.0319 |
|  | HL | DB3 | Trillium-40 | 28.50385 | 31.04697 | -0.0759 |
|  | HL | DB2 | Trillium-40 | 28.5039 | 31.047 | -0.507 |
|  | HL | MAT | Trillium-40 | 27.0964 | 31.094 | 0.043 |
|  | HL | MATC | L4C | 27.2305 | 31.3457 | -0.26 |
|  | HL | SLM | Trillium-240 | 25.2123 | 31.4916 | -0.182 |

| **Table (A-3)** North Egypt (Station Correction) | | | | | | |
| --- | --- | --- | --- | --- | --- | --- |
| **Network** | **Network Code** | **St. code** | **Sensor type** | **Longitude (E⸰)** | **Latitude (N°)** | **Station Correction (S)** |
| Helwan/ Egyptian national seismological network (ENSN) | HL | MSM | SS1 | 31.889 | 22.8814 | -0.0961 |
|  | HL | MABD | Trillium-240 | 32.3258 | 22.9726 | -0.0615 |
|  | HL | NNAL | Trillium-40 | 32.6647 | 23.2931 | -0.0527 |
|  | HL | NWAL | Trillium-40 | 32.5779 | 23.3829 | 0.102 |
|  | HL | NWKL | Trillium-40 | 32.44888 | 23.41309 | 0.0544 |
|  | HL | NGAL | Trillium-40 | 32.7324 | 23.4192 | -0.00749 |
|  | HL | NGMR | Trillium-40 | 32.40756 | 23.52169 | 0.0174 |
|  | HL | KSR | Trillium-40 | 33.0872 | 23.6105 | 0.413 |
|  | HL | NSKD | Trillium-40 | 32.386 | 23.661 | -0.232 |
|  | HL | NGRW | Trillium-40 | 32.7912 | 23.6684 | 0.226 |
|  | HL | NNMR | Trillium-40 | 32.5621 | 23.7379 | -0.234 |
|  | HL | NAHD | Trillium-40 | 32.778 | 23.8022 | 0.236 |
|  | HL | BRNS | Trillium-240 | 34.1143 | 23.8559 | -0.107 |
|  | HL | NMAN | Trillium-40 | 33.0737 | 23.9169 | 0.397 |
|  | HL | NKUR | Trillium-40 | 32.6514 | 24.0042 | -0.196 |
|  | HL | NGWR | Trillium-40 | 32.9535 | 24.2495 | 0.121 |
|  | HL | DK2 | Trillium 120 | 28.9546 | 24.3195 | -0.155 |
|  | HL | NEDF | Trillium 120 | 32.9564 | 24.8903 | -0.159 |
|  | HL | MRS | Trillium 120 | 34.8387 | 25.0131 | -0.00785 |
|  | HL | NMRS | Trillium-40 | 34.868 | 25.063 | 0.0161 |
|  | HL | NADB | Trillium-240 | 34.5021 | 25.3405 | 0.365 |
|  | HL | GTR | SS1 | 30.5595 | 25.5096 | -0.0653 |
|  | HL | DK1 | Trillium 120 | 29.4028 | 25.5432 | -0.0912 |
|  | HL | HRG | Trillium 120 | 33.6081 | 27.0517 | 0.158 |
|  | HL | FRF | Trillium-240 | 28.3105 | 27.1484 | 0.0393 |
|  | HL | SUT | T 120C | 31.5626 | 27.3967 | -0.0765 |
|  | HL | TAMR | Trillium-240 | 30.9175 | 27.6821 | 0.14 |
|  | HL | SH2 | Trillium-240 | 34.0833 | 27.8817 | 0.285 |
|  | HL | SHR | Trillium 120 | 34.2301 | 27.8947 | 0.105 |
|  | HL | GRB | SS1 | 32.7859 | 28.2705 | 0.328 |
|  | HL | TR2 | Trillium compact | 33.7227 | 28.3853 | 0.41 |
|  | HL | KAT | Trillium-40 | 33.9928 | 28.5229 | 0.232 |
|  | HL | NBNS | Trillium-40 | 31.2945 | 28.6226 | 0.029 |
|  | HL | RDS | SS1 | 33.2975 | 28.712 | 0.621 |
|  | HL | DHB | L4C | 34.6188 | 28.7221 | 0.523 |
|  | HL | BNS | Trillium-240 | 31.2126 | 28.9517 | -0.0097 |
|  | HL | RYAN | Trillium 120 | 30.27754 | 29.08251 | -0.0358 |
|  | HL | BST | Trillium-40 | 34.7327 | 29.2166 | -0.399 |
|  | HL | SWA | Trillium-240 | 25.4556 | 29.2432 | -0.116 |
|  | HL | ZAF | Trillium 120 | 32.5487 | 29.2819 | -0.252 |
|  | HL | ZNM | SS1 | 32.8752 | 29.3761 | -0.139 |
|  | HL | GLL | SS1 | 31.7081 | 29.5772 | 0.644 |
|  | HL | SAF | Trillium 120 | 31.5538 | 29.6187 | -0.087 |
|  | HL | NAT | Trillium 120 | 30.6172 | 29.6329 | -0.289 |
|  | HL | AYT | SS1 | 31.153 | 29.704 | 0.0545 |
|  | HL | MYD | L4C | 30.8009 | 29.7958 | 0.55 |
|  | HL | HLW | SS1 | 31.3432 | 29.8585 | 0.66 |
|  | HL | SQR | SS1 | 31.1959 | 29.8813 | 0.0792 |
|  | HL | KOT | STS-2 | 31.8292 | 29.9276 | 0.0462 |
|  | HL | NKL | Trillium-240 | 33.9804 | 29.9293 | 0.0934 |
|  | HL | HAG | SS1 | 32.099 | 29.953 | -0.195 |
|  | HL | BRG | SS1 | 29.8393 | 30.5743 | 0.0209 |
|  | HL | DB3 | Trillium-40 | 28.50385 | 31.04697 | -0.0248 |
|  | HL | DB2 | Trillium-40 | 28.5039 | 31.047 | -0.287 |
|  | HL | MAT | Trillium-40 | 27.0964 | 31.094 | 0.169 |
|  | HL | MATC | L4C | 27.2305 | 31.3457 | -0.0781 |
|  | HL | SLM | Trillium-240 | 25.2123 | 31.4916 | -0.0349 |

| **Table (A-4)** South Egypt (Station Correction) | | | | | | |
| --- | --- | --- | --- | --- | --- | --- |
| **Network** | **Network Code** | **St. code** | **Sensor type** | **Longitude (E⸰)** | **Latitude (N°)** | **Station Correction (S)** |
| Helwan/ Egyptian national seismological network (ENSN) | HL | AND | SS1 | 31.5307 | 22.1223 | 0.216 |
|  | HL | KFR | SS1 | 31.3047 | 22.5891 | -0.0102 |
|  | HL | MSM | SS1 | 31.889 | 22.8814 | -0.0863 |
|  | HL | MABD | Trillium-240 | 32.3258 | 22.9726 | 0.15 |
|  | HL | SHL | SS1 | 35.3999 | 23.1067 | 0.306 |
|  | HL | SHG | SS1 | 31.6576 | 23.2655 | 0.0942 |
|  | HL | NNAL | Trillium-40 | 32.6647 | 23.2931 | -0.0429 |
|  | HL | NWAL | Trillium-40 | 32.5779 | 23.3829 | -0.00376 |
|  | HL | NWKL | Trillium-40 | 32.44888 | 23.41309 | 0.115 |
|  | HL | NGAL | Trillium-40 | 32.7324 | 23.4192 | -0.132 |
|  | HL | NGMR | Trillium-40 | 32.40756 | 23.52169 | 0.0486 |
|  | HL | NSKD | Trillium-40 | 32.386 | 23.661 | -0.125 |
|  | HL | KRL | Trillium-40 | 32.7211 | 23.6634 | 0.0269 |
|  | HL | NKRL | Trillium-40 | 32.7211 | 23.6634 | 0.402 |
|  | HL | NGRW | Trillium-40 | 32.7912 | 23.6684 | 0.168 |
|  | HL | NMR | Trillium-40 | 32.5626 | 23.734 | -0.259 |
|  | HL | NNMR | Trillium-40 | 32.5621 | 23.7379 | -0.315 |
|  | HL | AHD | Trillium-40 | 32.751 | 23.7474 | 0.151 |
|  | HL | NAHD | Trillium-40 | 32.778 | 23.8022 | 0.13 |
|  | HL | BRNS | Trillium-240 | 34.1143 | 23.8559 | -0.0664 |
|  | HL | NMAN | Trillium-40 | 33.0737 | 23.9169 | 0.113 |
|  | HL | NKUR | Trillium-40 | 32.6514 | 24.0042 | -0.127 |
|  | HL | NGWR | Trillium-40 | 32.9535 | 24.2495 | 0.199 |
|  | HL | DK2 | Trillium 120 | 28.9546 | 24.3195 | -0.445 |
|  | HL | AGS | Trillium 120 | 34.9866 | 24.3794 | 0.513 |
|  | HL | BRS |  | 30.4169 | 24.70944 | -0.13 |
|  | HL | MRS | Trillium 120 | 34.8387 | 25.0131 | -0.0991 |
|  | HL | EDF | Trillium-40 | 33.1818 | 25.0945 | -0.174 |
|  | HL | NADB | Trillium-240 | 34.5021 | 25.3405 | 0.202 |
|  | HL | KRG | SS1 | 30.4985 | 25.5032 | 0.0736 |
|  | HL | DK1 | Trillium 120 | 29.4028 | 25.5432 | 0.187 |
|  | HL | QSR | Trillium 120 | 34.3871 | 25.7554 | 0.572 |
|  | HL | SFG | Trillium 120 | 33.9292 | 26.5675 | 0.293 |
|  | HL | HRG | Trillium 120 | 33.6081 | 27.0517 | 0.187 |
|  | HL | FRF | Trillium-240 | 28.3105 | 27.1484 | -0.0137 |
|  | HL | TAMR | Trillium-240 | 30.9175 | 27.6821 | 0.283 |
|  | HL | SH2 | Trillium-240 | 34.0833 | 27.8817 | 0.232 |
|  | HL | SHR | Trillium 120 | 34.2301 | 27.8947 | 0.32 |
|  | HL | GRB | SS1 | 32.7859 | 28.2705 | 0.29 |
|  | HL | KAT | Trillium-40 | 33.9928 | 28.5229 | 0.551 |
|  | HL | NBNS | Trillium-40 | 31.2945 | 28.6226 | 0.0625 |
|  | HL | DHB | L4C | 34.6188 | 28.7221 | 0.782 |
|  | HL | BST | Trillium-40 | 34.7327 | 29.2166 | -0.314 |
|  | HL | SWA | Trillium-240 | 25.4556 | 29.2432 | -0.0225 |
|  | HL | ZNM | SS1 | 32.8752 | 29.3761 | -0.176 |
|  | HL | GLL | SS1 | 31.7081 | 29.5772 | 0.294 |
|  | HL | KOT | STS-2 | 31.8292 | 29.9276 | 0.0546 |
|  | HL | NKL | Trillium-240 | 33.9804 | 29.9293 | 0.146 |
|  | HL | HAG | SS1 | 32.099 | 29.953 | 0.00747 |

### A3: Frequency-magnitude distribution

**1-Mediterranean Sea:**


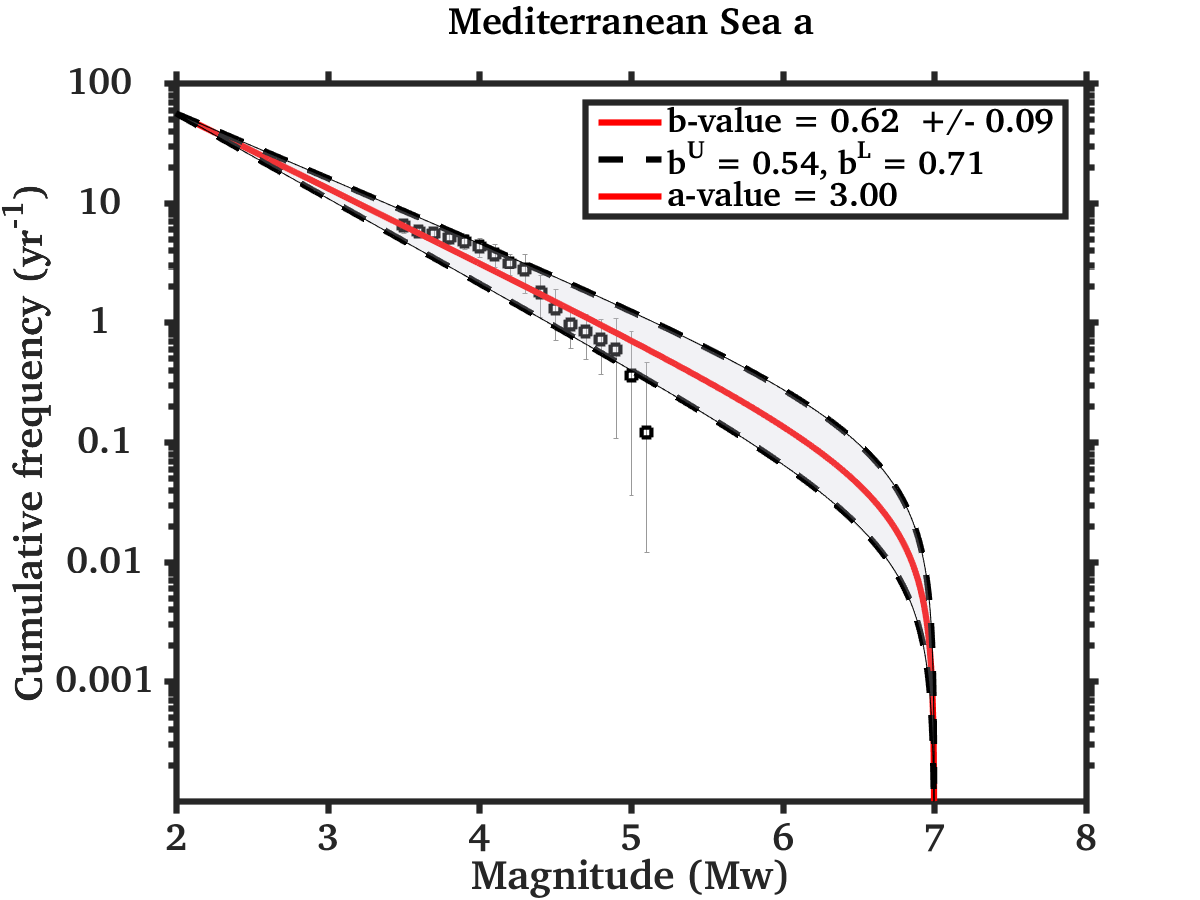

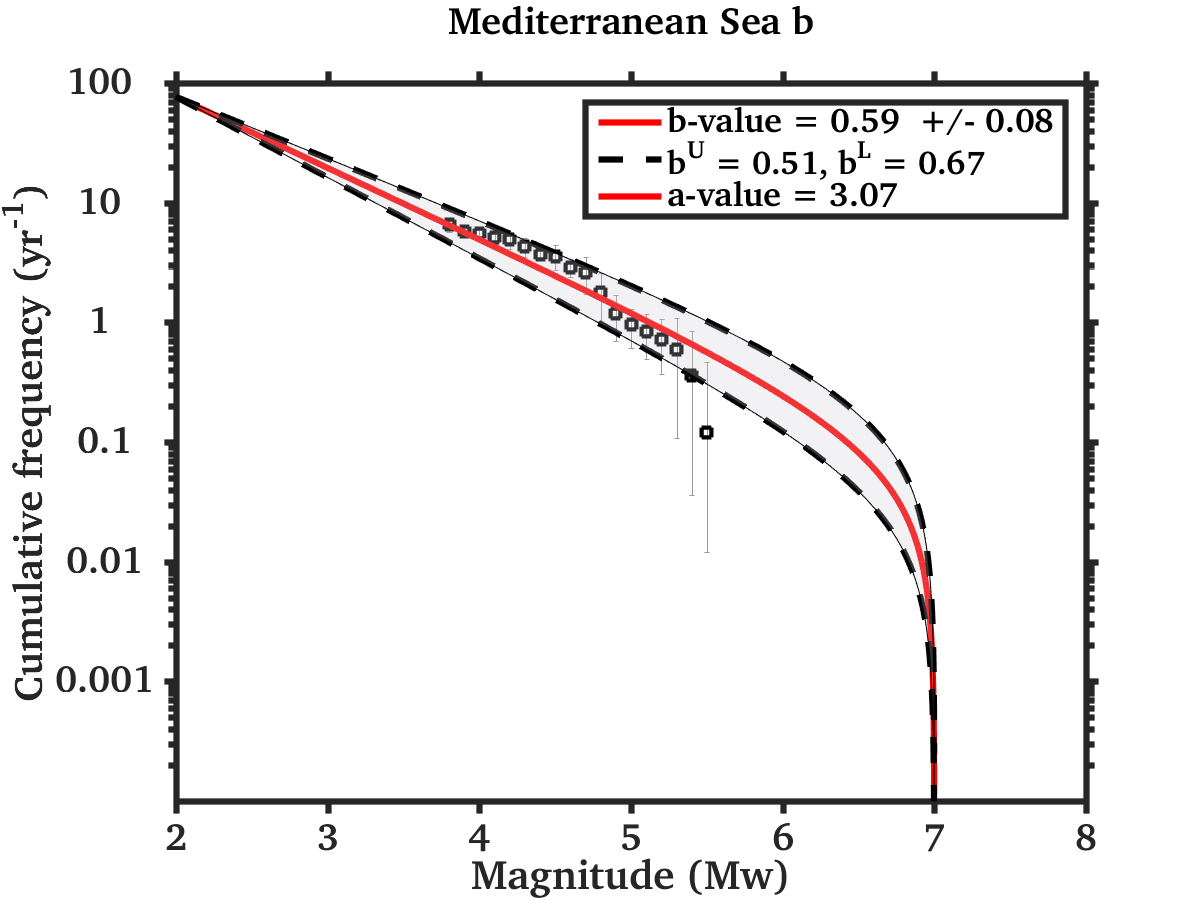


Figure (A-2): The Gutenberg-Richter distribution for the Mediterranean Sea Region based on Old ML (a) and Newly obtainned ML (b)

**2. Red Sea**


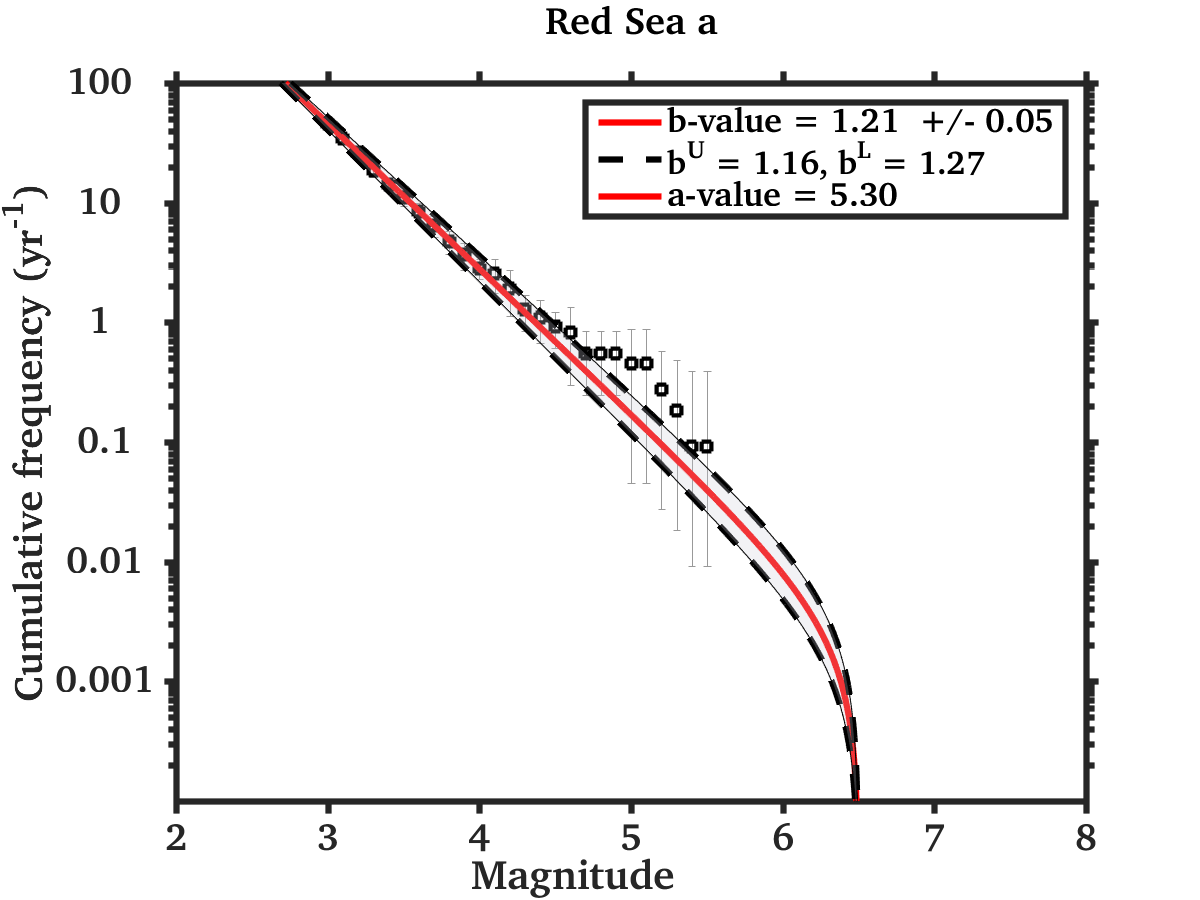


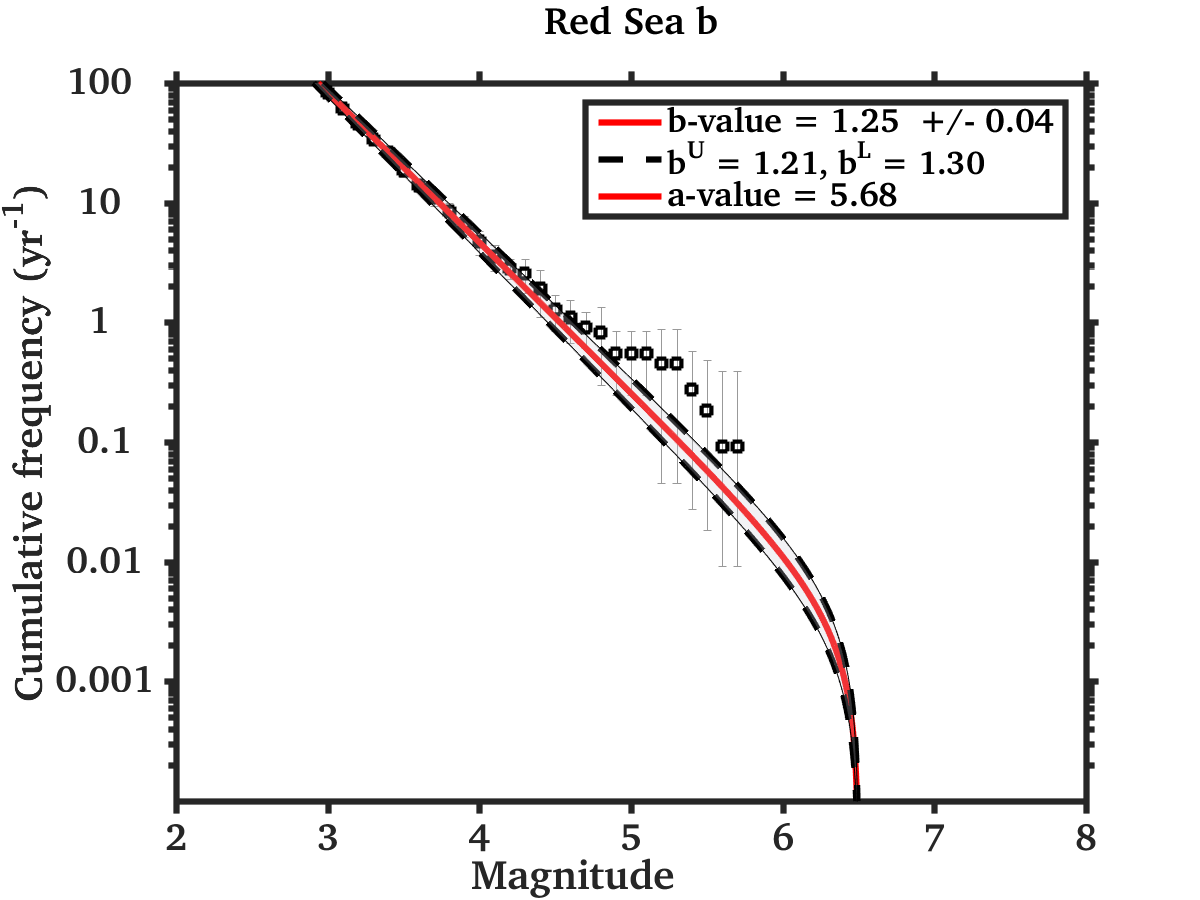


Figure (A-3): The Gutenberg-Richter distribution for the Red Sea Region based on Old ML (a) and Newly obtained ML (b).

**3. North Egypt:**


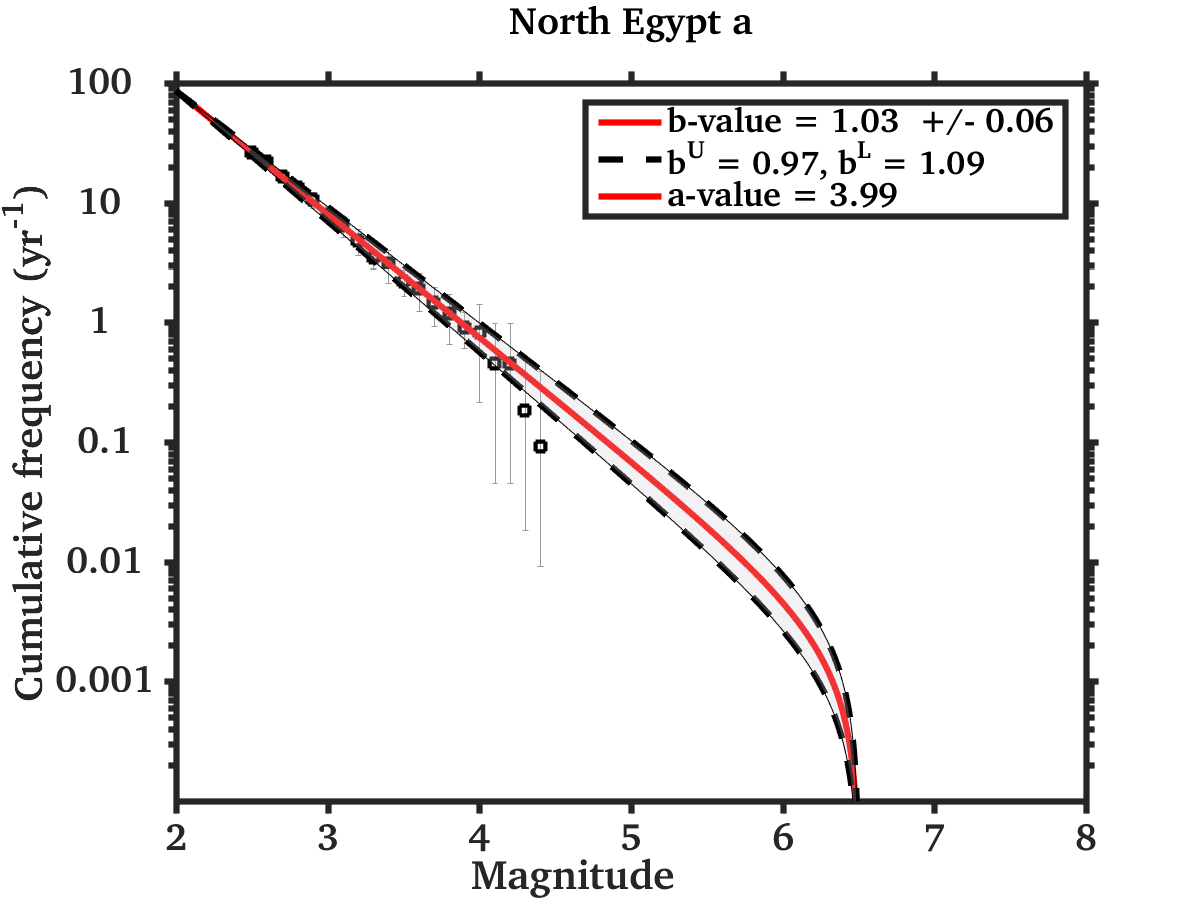


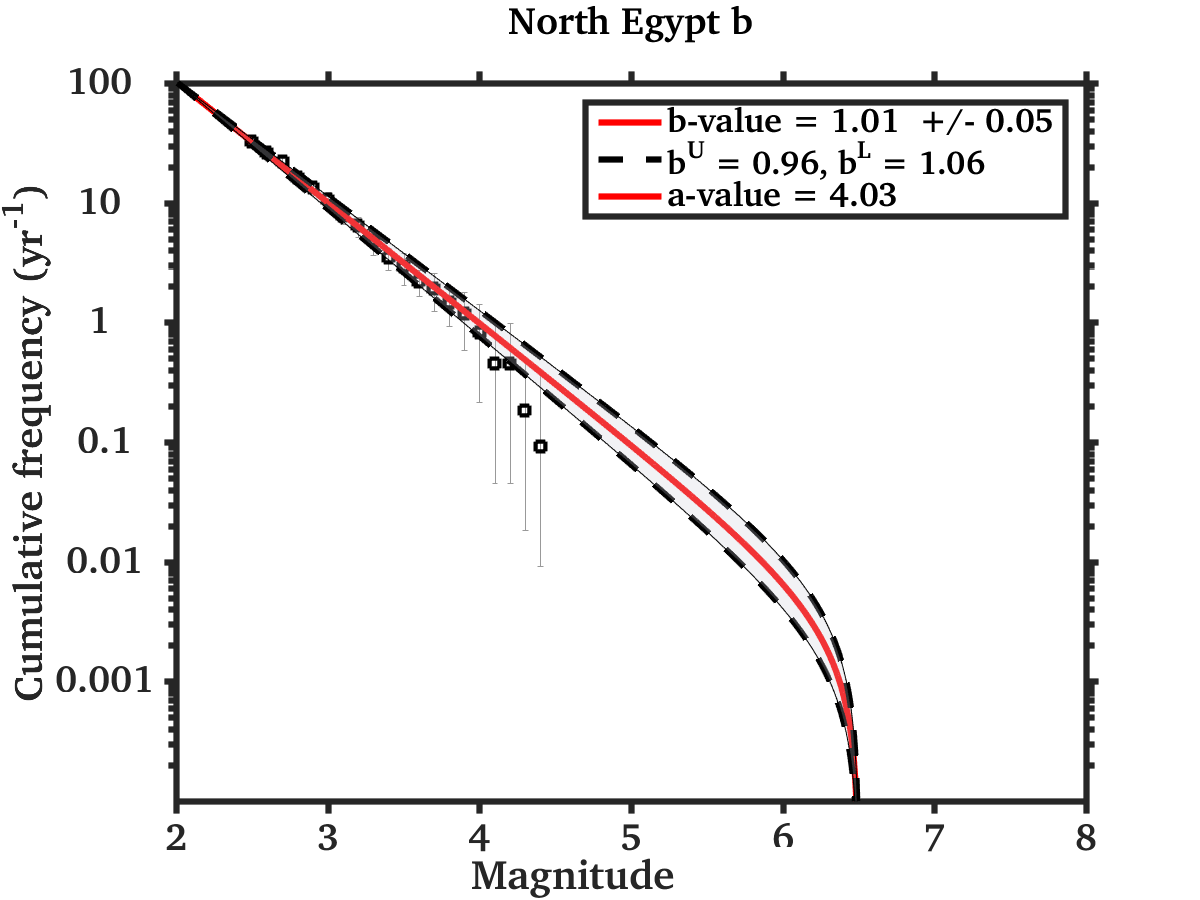


Figure (A-4): The Gutenberg-Richter distribution for the North Egypt Region based on Old ML (a) and Newly obtained ML (b).

**4. South Egypt**


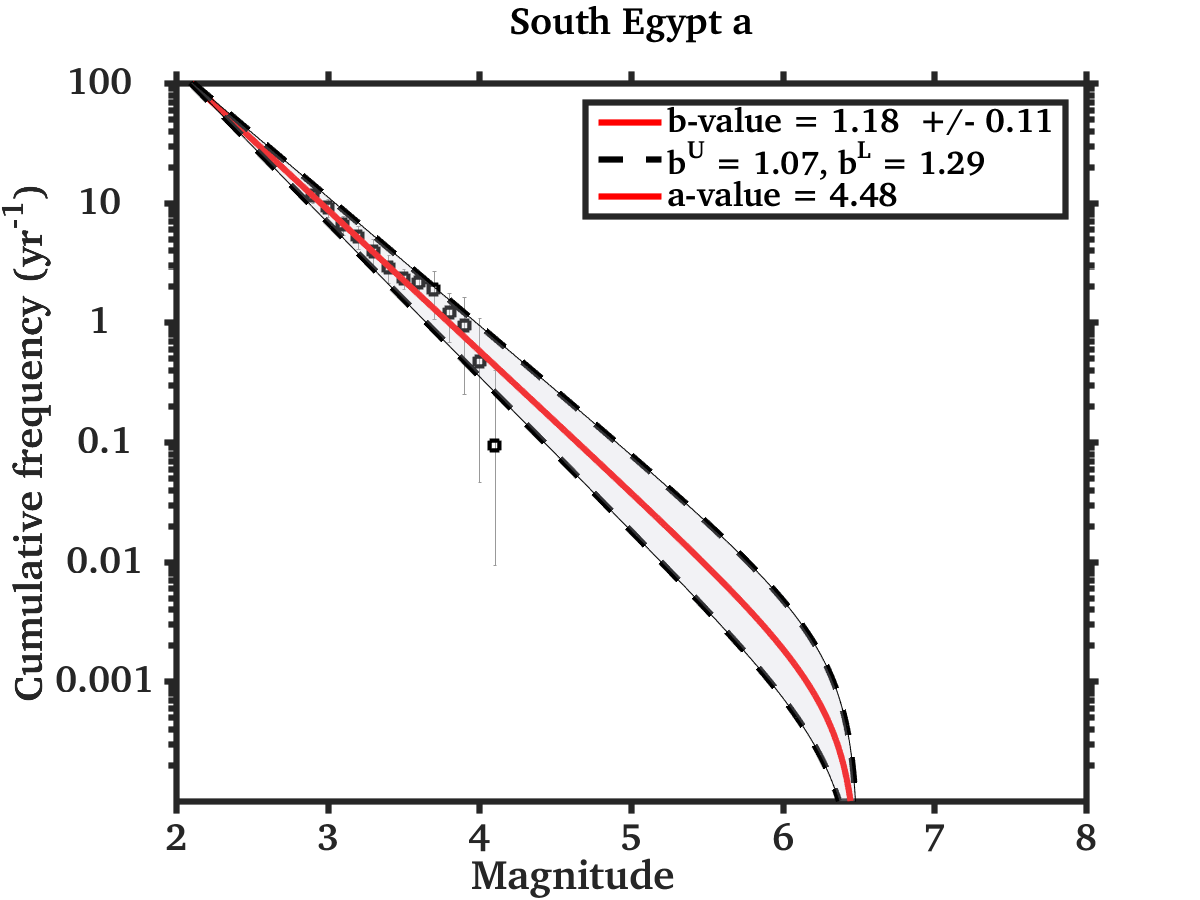


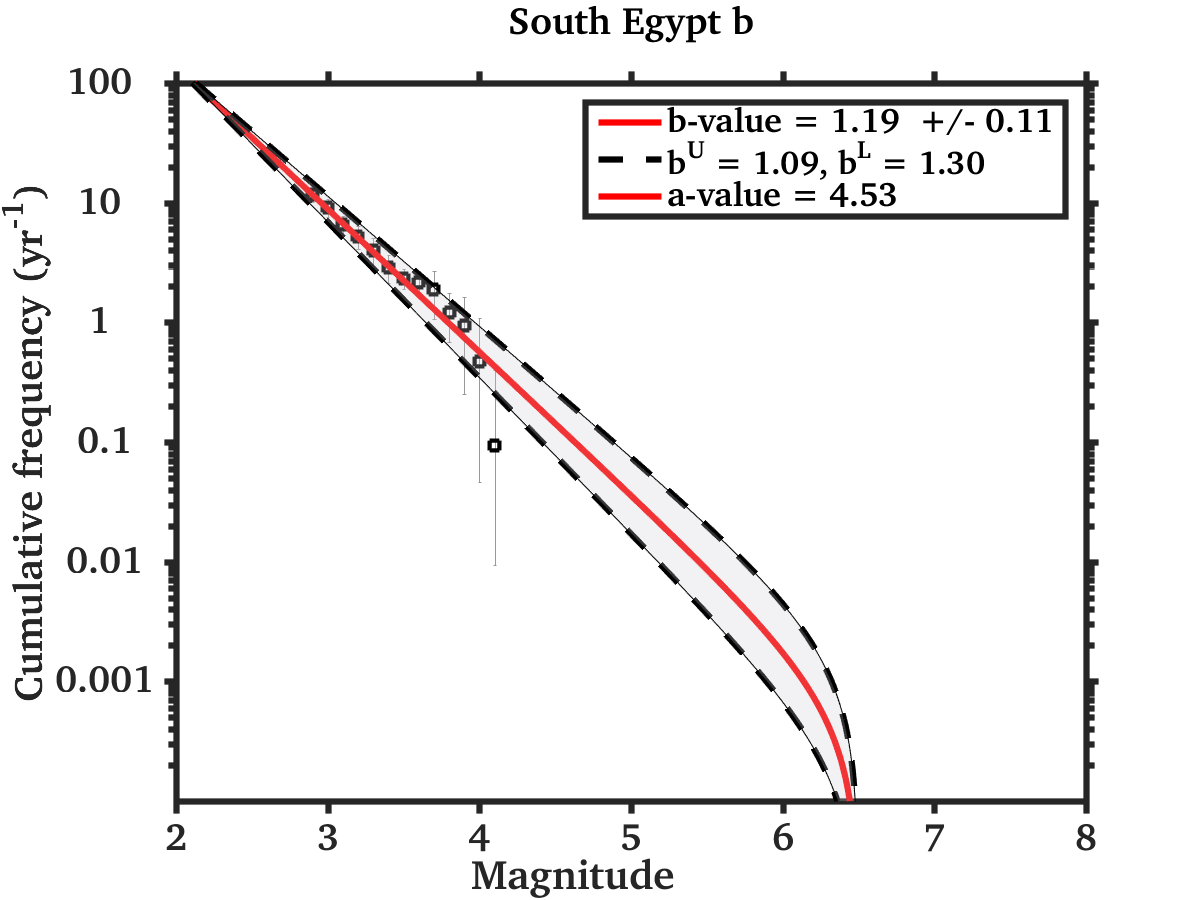


Figure (A-5): The Gutenberg-Richter distribution for the South Egypt Region based on Old ML (a) and Newly obtained ML (b).
